# If graffiti changed anything, it would be illegal. The influence of political graffiti on the perception of neighborhoods and intergroup attitudes

**DOI:** 10.3389/fpsyg.2023.1098105

**Published:** 2023-07-13

**Authors:** Claas Pollmanns, Frank Asbrock

**Affiliations:** Department of Psychology, Chemnitz University of Technology, Chemnitz, Germany

**Keywords:** political graffiti, political orientation, social cohesion, social norms, dose–response relationship

## Abstract

In a series of three studies (total *N* = 956), we examined how political graffiti, which serves as a representation of prevailing social norms, influences the evaluation of social cohesion and neighborhood inhabitants depending on the individuals political orientation. In line with our hypothesis, results of Study 1 (*N* = 199) indicated that individuals tended to express more positive evaluations of the social cohesion within a neighborhood when the political graffiti aligns with their own political orientation. Conversely, when confronted with counter-attitudinal political graffiti, participants reported lower evaluations of social cohesion. In Study 2, a sensitive scale to assess social cohesion was developed. Study 3 (*N* = 550) investigated the dose–response relationship of right-wing political graffiti and replicated the results from Study 1. Consistent with our hypotheses, even a minimal presence of right-wing graffiti exerted a significant impact on participants’ evaluations of the neighborhood and interacted with the participants political orientation. Taken together, our studies shed light on the crucial role of the individuals’ own political orientation for the evaluation of neighborhoods and their inhabitants. Furthermore, we offer insights into how these perceptions may influence intergroup attitudes toward foreigners living in Germany. The implications of our findings are highly relevant to ongoing discussions surrounding social norms within neighborhoods. By highlighting the significance of political graffiti as a representation of social norms, our research contributes to a deeper understanding of the dynamics at play in evaluating neighborhoods and their social fabric.

## Introduction

Political graffiti has a rich history spanning thousands of years. Archaeologists have discovered examples of political graffiti in the remains of ancient Greece and Rome, which aimed to ridicule political opponents or express support for specific political attitudes or positions (Baird and Taylor, 2010). Such graffiti often comprised written messages and symbols that conveyed various political viewpoints. Remarkably, even in contemporary times, the urban landscape continues to be marked with political graffiti that reflect ongoing societal discourses.

In research, the state of the environment has been recognized as a reference point for assessing social norms, safety, the evaluation of the residents (Kelling and Wilson, 1982; Cialdini et al., 1991; O’Brien and Wilson, 2011; Salesses et al., 2013). Kelling and Wilson (1982) in their Broken Windows Theory suggested that people form their perceptions of a specific neighborhood based on cues of social and physical disorder in the environment. Research suggests that the appearance of physical disorders signals that social control and the enforcement of social norms in a neighborhood have broken down and results in a higher likelihood that residents perceive their neighborhood as less safe (Alves Diniz and Stafford, 2021; Miethe and Venger, 2021). Both aspects have been shown to lead to feelings of stress (Hill and Angel, 2005), lower quality of life and health (Kawachi et al., 1999), and inhibit social cohesion among residents (Ross and Jang, 2000).

Graffiti serves as one of these cues and is considered a manifestation of physical disorder. However, despite extensive research conducted on the effects of physical disorder on these variables (for a recent Meta-analysis, refer to: O’Brien et al., 2019), the evidence regarding the isolated influence of graffiti, separate from other forms of physical disorder remains inconclusive (for a recent review, see: Alves Diniz and Stafford, 2021). Plausible explanations for these inconclusive findings may stem from the quantity of graffiti in a neighborhood and the mostly correlative study designs that lack the ability to experimentally control the confounders’ influence. For example, when examining prior studies investigating the impact of (environmental) stimuli on individuals, research has consistently suggested that the number of cues influences the responses of individuals (Cialdini et al., 1991; Arendt, 2015; Bergquist et al., 2021).

Moreover, previous scholars posit that physical disorder, including graffiti specifically, serves as an indicator of a neighborhood’s acceptance or lack of disapproval toward certain attitudes or behaviors (Kelling and Wilson, 1982; Cialdini et al., 1991). In that sense, graffiti is frequently used to mark the territory of particular groups or subcultures (Bloch and Phillips, 2020; Hughes et al., 2021) with specific codes and forms of representations (Bloch and Phillips, 2020; Alves Diniz and Stafford, 2021). This makes the physical environment a reflection of the enforcement of social norms and the overall social atmosphere within neighborhoods (Kelling and Wilson, 1982). In this case, the presence of political graffiti should be differentially appealing to individuals regarding their own political orientations.

In the current study, our primary objective was to examine the impact of political graffiti (specifically, those associated with left-wing and right-wing ideologies) on the evaluation of neighborhoods, the perceived level of social cohesion, and the evaluation of the residents within these neighborhoods. Additionally, we aim to address a previously unanswered question regarding the threshold of graffiti exposure required to elicit effects in individuals and propose a potential dose–response relationship (Arendt, 2015). Put differently, we investigated how much right-wing graffiti is needed to influence the perception of a neighborhood and how the effect changed with more graffiti in a neighborhood. Eventually we exploratory investigated the influence of right-wing political graffiti on the participants’ attitudes toward foreigners living in Germany. Our overall goal was to provide insights into the question if and how people are influenced in their attitudes by cues in their physical surroundings when navigating through the city.

### Social norms in the environment

Research on social norms has a long tradition in social psychology (Sherif, 1936; Asch, 1951; Sherif and Sherif, 1953). Social norms serve the purpose of reducing uncertainty within social contexts and providing behavioral guidance for both in-group and out-group members (Crandall et al., 2002). They are essentially “shared patterns of thought, feeling, and behavior” (Hogg and Reid, 2006, p. 8) that exist between members of a society or a group.

Despite the innate desire of individuals to comprehend and conform to the prevailing norms within their respective groups or communities, the perception of social norms can often be limited and prone to inaccuracies (Tankard and Paluck, 2016). Consequently, individuals form their understanding of norms based on observations and selective information obtained from their social environment. The motivation to align behavior with social norms originates from the need for group affiliation and the apprehension of potential rejection for deviating significantly from the established norm (Cialdini and Goldstein, 2004).

Early field studies conducted by Minard (1952), Reitzes (1953), and Kohn and Williams (1956) establishing the dependence of behavior on situational and local contexts. These studies posited that different locations create distinct normative contexts. In alignment with this notion, Crandall et al. (2002) suggested that “in certain circumstances, the expression of prejudice toward inappropriate targets is tolerated – in the locker room, at the poker table, or among close friends from the neighborhood where certain prejudices are tolerated” (p. 373). Building upon these insights, we contend that valuable insights into social norms and neighborhood dynamics can be drawn from the environment itself, in the here presented case specifically through the presence of political graffiti and environmental cues within urban spaces.

The condition of the environment has played a pivotal role in representing social norms in social science research (Kelling and Wilson, 1982; Cialdini et al., 1991). It inspired research that investigated the effect of the physical neighborhood conditions on various factors such as the perception of social cohesion (O’Brien and Wilson, 2011) or fear of crime. A recent review and meta-analysis however state that research methods in this field appear to be flawed and further research is needed (O’Brien et al., 2019). Our objective was to tackle this concern by conducting the present studies using established and controlled methodologies as well as pretested measurement scales in order to effectively address our research questions.

To address this concern, we use the *thin slices paradigm* (Ambady et al., 2000). This paradigm draws upon a wealth of research from various disciplines outside of psychology, demonstrating humans’ ability to form accurate judgments based on brief exposures to scenes or photographs. Notably, research has shown that people accurately assess other people’s emotions, intentions, or motives from photographs or short videos (Hall et al., 2016; Schmid Mast and Hall, 2018). In the context of neighborhood perception studies utilizing images conducted by O’Brien and Wilson (2011) or Salesses et al. (2013), participants evaluated the social dynamics of specific areas after examining photographs of neighborhoods. It was found that participants’ perceptions of social dynamics aligned with ratings provided by residents of the respective neighborhoods (O’Brien and Wilson, 2011). Furthermore, perceptions of safety and social status were found to correlate with actual crime rates and income levels within the areas (Salesses et al., 2013).

Finally, a recent replication of Cialdini et al. (1991) by Bergquist et al. (2021) showed that the amount of normative cues in a room increased the amount of intended behavior in participants, indicating a dose–response dependency of normative cues. To our knowledge, the theoretical framework provided by Cialdini et al. (1991) of normative cues in environments has not found much attention outside the realm of environmental psychology (Stok and de Ridder, 2019) and we aimed to apply it innovatively to political cues in the environment.

### Social cohesion in neighborhoods

The concept of social cohesion has been frequently utilized by researchers to characterize social dynamics within neighborhoods over the past decades, and periodic reviews have been conducted to assess the current state of the field (Friedkin, 2004; Schiefer and van der Noll, 2017). However, its extensive use across various research disciplines and policy applications has resulted in a lack of a precise definition and operationalization of this construct. Consequently critiques contend that social cohesion is a multi-dimensional construct that refers to several aspects such as trust, reciprocity, social ties, solidarity, shared values, and the evaluation of the inhabitants (Breidahl et al., 2018). It is also discussed to represent a form of homogeneity within a community (Wickes et al., 2013). Notably, early research indicate a strong correlation between social control (the ability to enforce social norms and act against threats to safety and security) and the concept of social cohesion, often combining them into single scales (Sampson and Raudenbush, 1999; O’Brien and Wilson, 2011; Collins et al., 2017).

However, emerging research indicates the utility of considering control and cohesion as distinct constructs (Wickes et al., 2013; Collins et al., 2017). Social control plays an important in managing the diverse values and attitudes held by heterogeneous groups within a neighborhood, given that inhabitants may vary in their beliefs. Consequently, it remains an open question how important homogeneity is to social cohesion and if diversity (in its broadest sense) poses a potential challenge to social cohesion on a societal level (Putnam, 2007; Ariely, 2014).

In relation to political diversity within neighborhoods, research conducted in the United States has reported an increase in political segregation over the last decades (Motyl et al., 2014; Motyl, 2016). A recent US study by Motyl et al. (2020) revealed a growing trend of individuals self-segregating into politically homogenous communities and selecting neighborhoods based on social cues. Interestingly, participants demonstrated a preference for communities that aligned with their own political beliefs, even in the absence of explicit information about the political composition of the community’s inhabitants. The authors concluded that people’s perceptions of communities may be influenced by subtle cues, indicating the role of implicit factors in shaping community choices.

### Attitudinal and counter-attitudinal information exposure

According to Festinger’s (1957) theory of cognitive dissonance, individuals experience a state of discomfort when confronted with messages or situations that contradict their expectations or attitudes. In response, they employ various strategies to alleviate this dissonance, such as selectively exposing themselves to confirming information, rejecting opposing viewpoints, or devaluating individuals holding opposing views (Stroud, 2014). This theory has found recent application in the study of political messages within news and media (Arceneaux et al., 2013; Camaj, 2019; Jean Tsang, 2019). It has been observed that individuals tend to prefer messages that align with their pre-existing political views, reinforcing their initial positions (Knobloch-Westerwick and Meng, 2011) and even leading them to adopt more extreme positions (Levendusky, 2013). A meta-analysis conducted on this topic provides further insights into the effects of political message exposure indicating that people prefer congenial over uncongenial information (*d* = 0.36) (Hart et al., 2009).

Further, political messages tap into intergroup dynamics, as defending specific political information becomes an expression of ingroup solidarity (Van Bavel and Pereira, 2018; Wojcieszak and Garrett, 2018; Dvir-Gvirsman, 2019). Consequently, when individuals encounter political information that aligns with their own political identity, it tends to reinforce political polarization and may even lead to negative sentiments toward the opposing group (Garrett et al., 2014; Gvirsman, 2014).

Similarly, studies on exposure to counter-attitudinal information suggests an even further political divide, as participants’ opinions become even more divergent after being exposed to information that challenges their pre-existing beliefs. This exposure activates their political identity, leading to increased ingroup solidarity and hostility toward the outgroup (Arceneaux et al., 2013; Nyhan and Reifler, 2015; Zhou, 2016). Nevertheless, recent research has raised doubts about the presence of this backlash effect (Guess and Coppock, 2020).

In light of these findings, we formulated the hypothesis that perceiving political graffiti that aligns with an individual’s political opinion would result in a more favorable evaluation of the neighborhood and its potential senders (i.e., neighborhood inhabitants), thereby reinforcing the initial opinion. Conversely, we proposed that encountering graffiti that challenges the perceiver’s opinion would lead to a devaluation of the potential senders but would also strengthen the individual’s initial position.

### The present studies

In the present series of studies, our objective was to examine the impact of political graffiti within urban neighborhoods on individuals’ perception of the neighborhood and their individuals attitudes. We propose that political graffiti can be regarded as a form of political messaging, representing prevailing social norms that can either align or conflict with the recipients’ own attitudes. Therefore, we hypothesized an interaction effect between political graffiti and participants’ political orientation on the perception of social dynamics within a neighborhood. In Study 1 we postulate that exposure to political graffiti should tap into intergroup relations and evoke a positive or negative evaluation of the potential sender depending on the participant’s political orientation. In Study 2, we developed a robust and reliable measurement scale to thoroughly investigate this effect. Furthermore, in Study 3 we explored the possibility of a dose–response relationship of the effects of right-wing graffiti. Specifically, we anticipated that as the number of graffiti increased, the effect on individuals’ perceptions would also intensify.

Additionally, we conducted an exploratory analysis to examine the influence of graffiti on attitudes toward foreigners, aiming to assess whether our stimulus material had the ability to impact political attitudes related to the content of the graffiti.

All data and Supplementary materials can be retrieved from the OSF repository (https://osf.io/2cmwr/). All studies were preregistered (Preregistrations for each study can be found in the osf repository). In each study, we report how we determined our sample size, all data exclusions (if any), all manipulations, and all measures in the study (Simmons et al., 2012).

## Study 1

Study 1 constituted the initial examination of our hypothesis concerning the interaction between perceived political cues and individuals’ political orientation. More specifically, we hypothesized the following: (H1) Participants exposed to graffiti in the experimental groups with graffiti (independent variable), would rate our dependent variable *social cohesion* lower than the control group without graffiti. (H2) This evaluation would be moderated by participants own political orientation, such that individuals with a more left-wing orientation would rate pictures containing leftwing graffiti higher and pictures containing rightwing graffiti lower. (H3) Conversely, individuals with a rightist political orientation would mirror this pattern of evaluation as described in (H2).

### Method

#### Participants

A statistical power analysis with G*Power (medium effect size; alpha = 0.05; power = 0.80; 3 groups) estimated a minimum sample size of *N* = 156. We recruited a German sample of *N* = 232 participants over prolific.co. After excluding participants who had not correctly answered our control and accuracy items or failed to complete the survey, we remained with *n* = 199 participants. The mean age of the sample was 39 years (*SD* = 11.88) and 39.2% were women. The distribution of political orientation had a positive skew (skew = 0.48). Most participants identified as political Central (*n* = 63) and or oriented toward the political left (*n* = 106); only 40 identified as political right from the center.

#### Material

**Figure 1:**
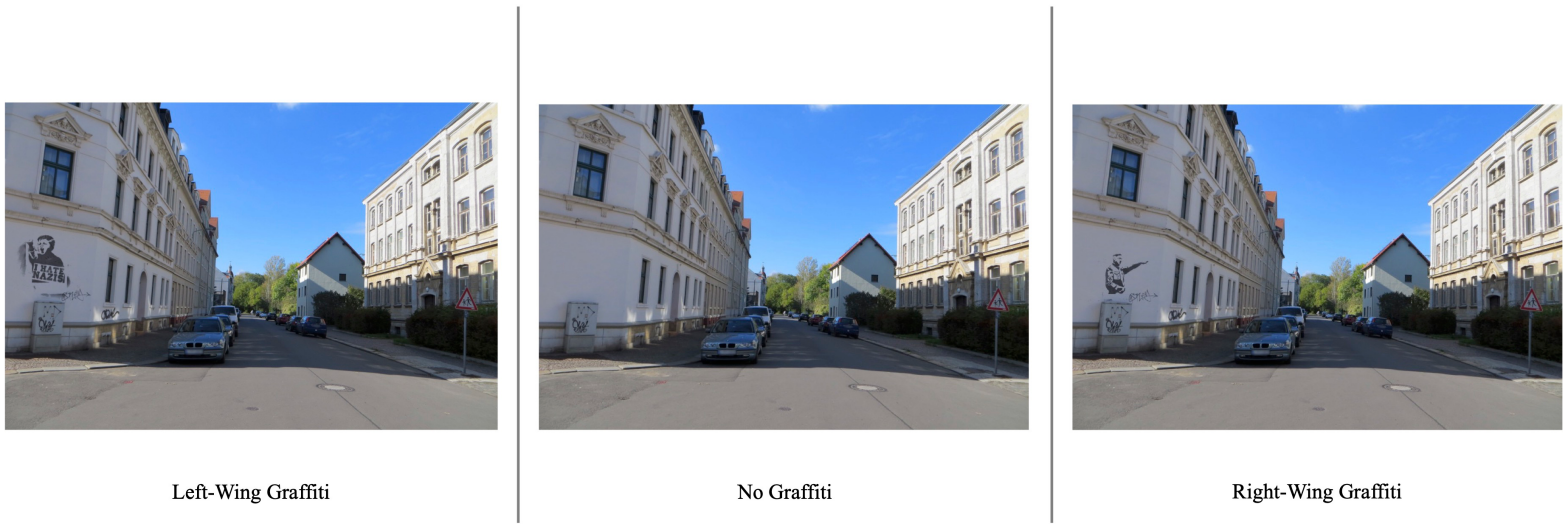
Example of a triplet of manipulated pictures.

In the picture selection process, we chose 10 images captured in a middle-class neighborhood under clear weather conditions. To ensure consistency and minimize potential biases, we digitally manipulated the pictures by removing street names, car license plates, pedestrians, and random graffiti. No visible indicators of the neighborhood’s ethnic or cultural diversity were included in the images. Next, we created three versions for a total of nine manipulated triplets. Digital additions of existing graffiti slogans or signs were placed uniformly in predetermined locations across all manipulated images. An example can be seen in Figure 1. To ensure the integrity and quality of the modified pictures, we sought feedback from 10 colleagues who examined and rated the images for any noticeable or suspicious manipulations. Pictures were refined accordingly. Eventually, the triplets consisted of: (1) right-wing and anti-migration cues representing the right-wing condition, (2) leftist and pro-migration cues representing the left-wing condition, and (3) a control condition without any graffiti or cues. All study materials can be found in the Supplementary materials S1.

#### Design

In an online study utilizing a between-subjects design with one factor, participants were randomly assigned to one of three conditions. To prevent biases against certain cities or regions, the instructions deliberately omitted any mentioning of a particular location. Subsequently, participants first saw a neutral test picture to avoid anchoring effects. Afterward, each participant saw nine pictures containing three pictures from the right-wing, left-wing, and neutral manipulation. Within the three conditions, the pictures were ordered and selected so that a between-subject comparison for the individual triplets (neutral, left-wing graffiti, right-wing graffiti) was possible. For programming constraints, we were unabley to fully randomize the order and valence of the pictures. Consequently, the pictures were shown in a predetermined order. Each picture was displayed for 10 s followed by a separate page featuring a thumbnail of the picture along with the assessment of our dependent variables. Upon completion of the experiment, participants were asked to provide information regarding their age, gender, and political orientation and an accuracy item as part of the post-study questionnaire.

#### Measures

The dependent variable to measure the participants’ perception of the neighborhood’s *social cohesion* consisted of three items from Sampson and Raudenbush (1999) and O’Brien and Wilson (2011), focusing on the perception of the people living in this area: (“*People around here are willing to help their neighbors,” “People in this neighborhood can be trusted,*” and “*There are adults in this neighborhood that children can look up to.*”) Items were rated on a 7-point Likert scale (1 = *strongly disagree*; 7 = *strongly agree*). The *warmth* of the neighborhood’s inhabitants was assessed with the semantic differential *cold – warm* on an 11-point Likert scale (Fiske et al., 2007). Finally, participants were asked to rate their perception of the neighborhood on the semantic differentials *orderly – disorderly* and *safe – unsafe* measured on an 11-point Likert scale adapted from O’Brien and Wilson (2011).

#### Manipulation check

Participants answered a manipulation check asking for the inhabitants’ left/right orientation in the depicted picture answered on an 11-point Likert scale. The item read *“What do you think is the political orientation of people living in this neighborhood?”* (1 = *very leftist*; 11 = *very rightist*).

#### Political orientation and demographics

The moderator political orientation was assessed using a 7-point Likert scale ranging from 1 (*left*) to 7 (*right*) at the end of the survey.

### Results and discussion

Detailed analysis of each dependent variable can be found in the Supplementary materials S1. As noted in our preregistration, previous research has indicated a high correlation among our dependent variables, which sometimes led to the use of a composite measure of social cohesion (Sampson and Raudenbush, 1999; O’Brien and Wilson, 2011; Kim and Kawachi, 2017). An exploratory factor analysis (EFA) with oblimin rotation revealed single-factor solutions over all items for all pictures, explaining 58–67% of the variance. Accordingly, we collapsed all items into composite scales. The internal consistency of these scales was high, with Cronbach’s alpha ranging from 0.89 to 0.94.

Prior to our main analysis, we conducted an analysis of variance (ANOVA) to examine the effect of the condition (independent variable) on participants’ responses to the manipulation test item, which asked about the perceived political orientation of the people living in the depicted neighborhood. This was followed by a *post-hoc* Tuckey honestly significant difference test (HSD) to compare group means. Our analysis revealed significant differences between all conditions for 7 out of the 9 manipulated pictures, providing evidence that participants accurately interpreted our manipulations as either right-wing or left-wing graffiti (See Supplementary materials S1).

**Figure 2:**
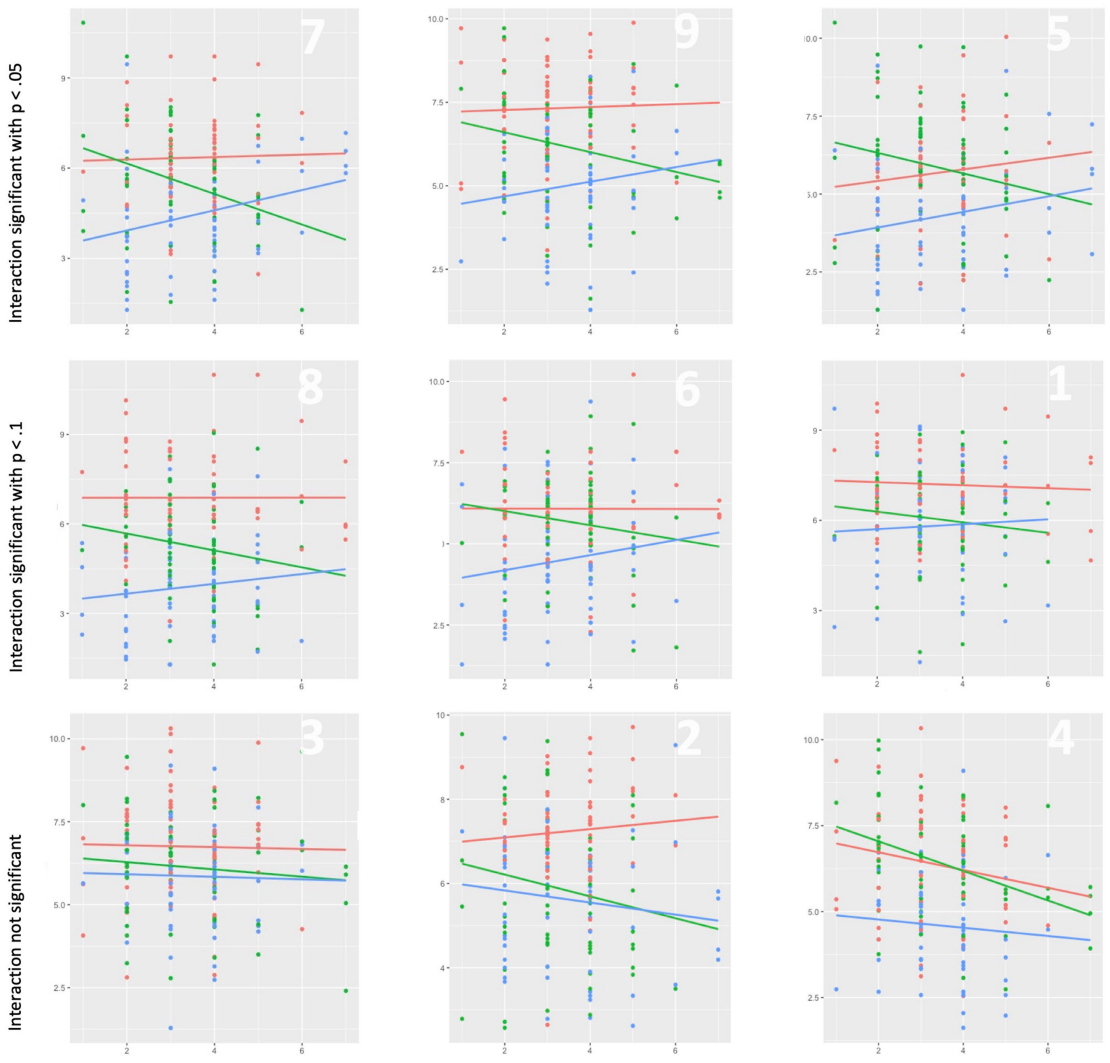
Plotted interactions from Study I. In the top right of each diagram the number of the triplets is indicated. On the *X*-axis the political orientation of the participants is plotted ranging from 1 (oriented toward the left) to 7 (oriented toward the right). On the *Y*-axis the dependent variable cohesion is plotted ranging from 1 to 11. The red regression slope is calculated from the neutral condition. The green regression slope is calculated from the left-wing condition. The blue regression slope is calculated from the right-wing condition.

In our main analysis, we tested our hypotheses in a moderated regression analysis, using the PROCESS Macro for R (Hayes, 2022). The variable political orientation was z-transformed before the analysis and we used Helmert coding for our multicategorial moderator (Hayes and Montoya, 2017). Overall, model fits *R*^2^ for all models ranged from 0.063 to 0.38 (*MR^2^* = 0.22, 95% CI [0.29, 0.16]). As expected, political graffiti influenced the evaluation of the neighborhood (H1), and we found the moderating effect of political orientation in 6 of the presented pictures. In three out of nine pictures the *R^2^*-Change for the interaction was significant (*p* < 0.05). Subsequently plotted the interaction for each picture to gain deeper understanding of the data. We observed the expected slope pattern in three additional pictures; however, the interaction did not reach statistical significance at convectional levels (all *p* < 0.08). In three pictures we did not find an interaction. We believe that this outcome can be attributed to a combination of factors, including the chosen pictures, failed manipulation tests, sample distribution, and sample size. Detailed visualization of the interaction plots are depicted in Figure 2. Notably, in those pictures where we did not find an interaction, the presence of graffiti alone had a negative effect on the overall evaluation, aligning with previous research on graffiti and its association with incivilities (Miethe and Venger, 2021).

A noteworthy limitation of our study, which bears significance for interpreting the results, is the non-normal distribution of our moderator variable, political orientation. The distribution showed positive skewness, indicating a greater number of participants identifying themselves as leftists. The relatively small number of right-leaning participants contributed to the observed results, whereby the right-wing conditions did not reach the same evaluation by rightist participants as the left-wing conditions did among leftist participants, thereby reducing the prominence of the interaction effect. Additionally, the generalizability of our findings is constrained by the relatively small magnitude of the interaction effects, stemming from the single exposure in each picture. This limitation could be attributed, for instance, to our deliberately chosen broad-range dependent variable.

Notwithstanding these limitations, our findings provide support for H1 across all pictures examined. Moreover, H2 and H3 were confirmed in 6 out of 9 pictures. Consequently, our results indicate that political graffiti holds appeal to those who align with the corresponding political ideas, resulting in a more positive perception of the neighborhood’s inhabitants and the potential senders of the conveyed messages within our experimental context. Building upon these initial findings, our subsequent studies aim to further refine our understanding of the impact of political graffiti.

## Study 2

To gain further confidence in the results of Study 1, our subsequent studies pursued two main objectives. Firstly, we sought to extend and refine our measurement for social cohesion beyond the scope of Study 1. Secondly, we aimed to replicate the observed effect and interaction using an alternative research design that would address the limitations identified in Study 1. By undertaking these steps, we aimed to strengthen the robustness and generalizability of our conclusions.

In Study 2, we specifically focused on refining the measurement of social cohesion. The concept of social cohesion has been widely used across various research domains, resulting in numerous scales attempting to capture its complexity. However, this has led to a lack of consensus on its multidimensionality, encompassing inter-group cooperation, attitudes toward neighbors, solidarity, norm enforcement, belonging, or neighborhood networks (Breidahl et al., 2018). Furthermore, existing social cohesion scales may be biased toward conservative values (Wickes et al., 2013) or may not be suitable for the German context. To address these limitations in previous research, our objective was to develop a measurement instrument capable of accurately assessing the perceived social dynamics of a neighborhood as perceived by external observers in the context of our experiments.

To accomplish this objective, we employed a multi-step approach. First, we selected or adapted items from established social cohesion scales (Sampson and Raudenbush, 1999; Lüdemann and Peter, 2007; O’Brien and Wilson, 2011; Collins et al., 2017; Kim and Kawachi, 2017). Additionally, we generated new items. During the item selection process, we selected items that fulfilled the following criteria: (a) They could be easily answered by people unfamiliar with the specific neighborhood under investigation. (b) They distinguish between *social cohesion*, *evaluation of inhabitants*, and *social control* which are common components in previously utilized scales, and (c) they would not be confounded with political attitudes. Eventually, we settled on 18 items as initial item Pool for the subsequent analysis, which can be reviewed in the Supplementary materials S2.

Based on prior research, we anticipated the emergence of two separate yet interrelated subscales: one for *social cohesion* and *social control* (Collins et al., 2017). Additionally, we expected the *evaluation of inhabitants* (e.g., trustworthy, friendly, etc.) to be a conceptually related construct. To validate our scales, we conducted exploratory and confirmatory factor analyses to assess their factor structure. Finally, we examined whether our scales could effectively detect differences between the presented neighborhoods by employing repeated measures ANOVA.^1^

### Method

#### Participants

To ensure sufficient statistical power for conducting confirmatory factor analyses (CFAs) with a conservative estimation of sample sizes (Myers et al., 2011) and considering our 18 items, we aimed to recruit a sample size of *N* > 180 participants. Participants were recruited via university mailing lists and social media groups targeting students. Participants were given the option to receive course credits for their participation or to enter a lottery for a chance to win vouchers. In total, we sampled responses from 254 German participants. To ensure data quality, we excluded participants who indicated on our control item (“*How thoroughly did you answer the questions in the questionnaire?*”), that they did not fill out the questionnaire thoroughly [rated >3 on a scale from 1 (very thoroughly) to 7 (not thoroughly at all)]. After applying this criterion, we remained with a sample of 212 participants. The mean age of this sample was 24.68 years (*SD* = 5.24) and 177 identifying as women (83.5%). Two participants did not indicate their gender.

#### Materials and study design

The design for Study 2 was adapted from previous research on neighborhood perceptions (O’Brien and Wilson, 2011). Each participant saw and rated three neighborhoods. We used pictures of neighborhoods that were taken on the same day as the pictures from Study 1. We selected two distinct neighborhood settings: one portraying a rural area and another depicting an urban neighborhood. Each of these neighborhoods was represented in six pictures. Additionally, a third neighborhood was represented by six neutral pictures (without graffiti) from Study 1, which had undergone a successful manipulation check and demonstrated an interaction effect. To minimize potential biases, we fully randomized the order of the pictures within each neighborhood, effectively controlling for any order effects. Each picture was displayed for a duration of 5 s. Thereafter all six pictures from one neighborhood were presented on one page as thumbnails, allowing participants to fill out the items for our dependent variables. This procedure was repeated for all presented neighborhoods.

#### Measures

If not otherwise stated, all items were measured on a 7-point Likert scale (1 = *strongly disagree*, 7 = *strongly agree*). All item wordings can be found in the Supplementary materials S2.

#### Social cohesion

We measure the first subscale of the social cohesion scale from Sampson and Raudenbush (1999) to assess *social cohesion* with 5 items. An example item read “*This is a close-knit neighborhood*.”

#### Social control

The items for the second subscale of the SC from Sampson and Raudenbush (1999) are confounded with conservative attitudes (Wickes et al., 2013) or do not apply to Germany. Therefore, we selected eight items from other SC scales (Lüdemann, 2005; Collins et al., 2017; Kim and Kawachi, 2017) and generated items that represent the ability to enforce norms and social control. An example item read “*Inhabitants would approach troublemakers*.” Items were assessed on a reversed 7-point Likert scale (1 = *strongly agree*; 7 = *strongly disagree*).

#### Evaluation of inhabitants

With 5 items we asked participants about their evaluation of the inhabitants using items from Kim and Kawachi (2017) we used in Study 1 and generated additional new items – all tapping into the communal dimension of stereotypes (Fiske et al., 2007). An example item read “*Most people in this area are unfriendly*.” (reversed).

### Analytical procedures

First, an exploratory principal factor analysis (EFA) with promax rotation was performed for the variables of the first neighborhood to identify suitable items. Subsequently, a confirmatory factor analysis (CFA) was performed using the data from areas 1, 2, and 3 to validate the identified factor structure. Model fit was assessed using the comparative fit index (CFI), Tucker–Lewis Index (TLI), and the root mean square error of approximation (RMSEA) following recommendations (Hu and Bentler, 1999). Internal reliability and scale means were calculated. Additionally, we conducted a repeated measure ANOVA to examine whether the scales were capable of detecting differences among the presented neighborhoods.

### Results and discussion

Detailed analysis, factor loadings, and item wording are reported in the Supplementary materials S2. In the first step of the analysis, all 18 items were included in the exploratory factor analysis (EFA). We used principal axis factor analysis with promax rotation and parallel factor extraction. In the second step, we dropped items with low or cross-loadings and selected items most suitable to represent the underlying constructs. This process resulted in a final set of 13 items that loaded onto three distinct factors. Factor one captured evaluations of the inhabitants and accounted for 14.6% of the variance. Factor two represented the ability to enforce social control and explained an additional 11.2% of the variance. The third factor explained 8.3% of the variance and represented the preceded social cohesion in the neighborhood. Together, these three factors accounted for 34.2% of the total variance.

In the subsequent step, we conducted confirmatory factor analysis (CFA) to validate the structure identified in the EFA for neighborhood 1 with each of the three neighborhoods. We made some adjustments during the CFA process. Firstly, we removed one item from the social cohesion scale due to its reverse scoring and strong residual covariances with other items in the same block. Secondly, we introduced residual covariances among items within each scale to account for the method factor of positive and negative wording. Eventually, we settled on four items for each dimension.

Overall fit indices for each neighborhood indicated a good fit (CFI > 0.96 TLI ≥ 0.95, RMSEA 0.4–0.6; RMSEA Upper 90% CI ≤ 0.08). However, during the analysis we observed that two items of the social cohesion factor had factor loadings bellow 0.5. Despite this, we decided to retain these items in the scale to maintain conceptual coverage. The results of the CFA are reported in the Supplementary materials for Study 2 (S2) and a summary is provided in Table 1.

**Table 1:** Confirmatory factor analysis fit indices for each tested neighborhood.

				RMSEA 90% CI	
	CFI	TLI	RMSEA	Lower	Upper
Neighborhood 1	0.96	0.95	0.04	0.00	0.06
Neighborhood 2	0.97	0.96	0.06	0.03	0.08
Neighborhood 3	0.97	0.96	0.05	0.02	0.07

Cronbach’s Alphas for the scales ranged from 0.56 to 0.89 and are reported in Table 2. Overall, we observed that some of the reversed worded items presented a minor challenge to scale validity and may have contributed to the slightly lower reliabilities. In particular, the variable indented to measure social cohesion showed mixed results in the factor analysis. However, it is important to note that this discrepancy may not necessarily stem from a statistical issue, but rather to the limited information conveyed in the pictures themselves. Since the questions posed to participants pertained complex social behavior, they might be not have been easily answered based solely on the visual stimuli provided at the pictures. Table 2 reports the correlations between the scales for further examination.

**Table 2:** Scale reliability, means, standard deviations, and intercorrelations between the scales.

				Correlations	
		*α*	*M* (*SD*)	1	2
Neighborhood 1	1. Evaluation of inhabitants	0.74	5.23 (0.90)		
	2. Social control	0.67	4.22 (1.10)	−0.01	
	3. Social cohesion	0.56	4.64 (0.72)	0.31^**^	0.13^*^
Neighborhood 2	1. Evaluation of inhabitants	0.80	5.33 (1.00)		
	2. Social control	0.89	5.04 (1.55)	0.53^**^	
	3. Social cohesion	0.83	5.49 (0.88)	−0.05	0.10
Neighborhood 3	1. Evaluation of inhabitants	0.75	4.46 (0.87)		
	2. Social control	0.79	3.75 (1.08)	0.34^**^	
	3. Social cohesion	0.61	3.85 (0.75)	0.66^**^	0.32^**^

^*^p < 0.05; ^**^p < 0.01.

Finally, we tested for perceived differences between the three neighborhoods conducting a repeated-measures ANOVA. Due to a violation of sphericity, we corrected degrees of freedom using Huynh-Feldt’s estimates of sphericity. The repeated measures ANOVA overall rated areas revealed significant results for all constructs: *η^2^* for social cohesion = 0.42, *F*(1.89, 398.69) = 266, *p* < 0.001; *η^2^* for evaluation of inhabitants = 0.15, *F*(1.98, 418.53) = 72, *p* < 0.001; *η^2^* for social control = 0.16, *F*(1.63, 344.25) = 68.6, *p* < 0.001. *Post hoc* tests indicated that all presented areas showed significant mean differences on our dependent variables except the evaluation of inhabitants between neighborhood 1 and neighborhood 2. *Post-hoc* tests can be found in the Supplementary materials S2. Overall, social cohesion exhibited the largest effect among the other dimensions in our repeated measure ANOVA, indicating that results were most clear on this facet of our dependent variable.

Taken together, the results from Study 2 indicated that our newly developed instrument exhibited favorable fit indices and yielded reliable scales. Additionally, it successfully captured participants’ varying perceptions of the social dynamics within three neighborhoods. Encouraged by these results, we decided to employ our measurement instrument in Study 3, where we sought to examine the impact of political graffiti on neighborhood perception with greater precision.

## Study 3

Our aim for Study 3 encompassed two primary aspects: Firstly, we aimed to replicate the interaction of political graffiti with political orientation observed in Study 1, utilizing a different design and a refined measurement instrument. Secondly, we sought to test a dose–response relationship for our manipulation (Cialdini et al., 1991; Arendt, 2015) by varying the number (e.g., the dose) of political graffiti across various conditions. Due to the large sample size and financial costs that come with such a design, we focused on the dose–response relationship solely with right-wing graffiti. We hypothesized that with the number of right-wing cues increased, participants express less social cohesion, social control, and a less positive evaluation of inhabitants (H4). However, we did not make further assumption about this relationship, as suggested by prior research conducted by Arendt (2015) and Cialdini et al. (1991) showed that the relationship must not be linear. Additionally, we anticipated that participants’ political orientation would moderate this dose–response effect, with a stronger negative impact for those leaning toward the left (H5) and a more positive impact for those leaning toward the right. Exploratory in nature, we also included a variable to assess how our manipulation would influence participants’ evaluation of foreigners residing in Germany (E1). The experiment was preregistered, and all Supplementary materials can be in the OSF.io repository.

### Method

#### Participants

We employed G*Power software to calculate our approximate sample size prior to Study 3. Based on the effect size obtained from Study 1, we calculated the sample size for testing the interaction for an ANOVA with *f* = 0.2 (small effect); Bonferroni-corrected alpha level = 0.0166; power = 0.8, *df* = 8; and 5 groups. The projected sample size was 483. To specifically examine the interaction between the control group and the first condition with one cue, where we expect to have the smallest effect, we calculated a sample size of 180 participants for *f* = 0.245 (largest effect found in study 1); alpha = 0.0167; power = 0.8; *df* = 1 and 2 groups. Ultimately, we settled on a projected sample size of 550; *n* = 110 per condition.

We recruited participants through prolific.co, ensuring adequate compensation. After removing participants who failed our attention test or indicated that they did not complete the questionnaire carefully, we remained with a sample of 554 participants. The mean age was 29.5 years (*SD* = 10.3); 272 were men, 269 were women, 8 were divers and 3 did not indicate their gender. The distribution of political orientation exhibited positive skewness (skew = 0.45) indicating a greater number of participants identifying with left-leaning orientations.

#### Material and procedure

We employed a between-subject design comprising one control condition and four experimental conditions. To present the neighborhoods, we utilized the same format as in Study 2, consisting of six pictures. However, for this study, only two neighborhoods were shown: one neutral neighborhood without any graffiti and one neighborhood with our manipulations. In the manipulated neighborhood, we varied the number of rightwing graffiti cues across the conditions as follows: control condition (0 cues), condition 1 (1 cue), condition 2 (2 cues), condition 3 (4 cues), and condition 4 (6 cues). We used those six pictures that stood the manipulation test in study 1 and showed the intended interaction pattern. After each presented neighborhood we asked participants to rate our dependent variables.

#### Measures

As dependent variables, we used the scales for *social cohesion, social control, and evaluation of inhabitants* we developed in Study 2. All items were answered on 7-point Likert scales from *1 = fully disagree/very unlikely* to *7 = fully agree/very likely*. Cronbach’s Alpha for the scales was: social cohesion = 0.77, social control = 0.75, and evaluation of inhabitants = 0.89.

#### Demographics and political orientation

Before presenting the conditions, we assessed the demographics of our sample. Our moderator *political orientation* was measured using a 7-point Likert scale ranging from 1 (*left*) to 7 (*right*).

For exploratory purposes, we added a *Feeling Thermometer* with two items (*r* = 0.78) (e.g., “*In general, how do you feel towards foreigners living in Germany*?) (Wright et al., 1997) after the presented neighborhoods and asked for attitudes toward immigrants on a slider from 1 to 100 (1 = *cold / very negative*; 100 = *warm / very positive*).

### Results and discussion

**Figure 3:**
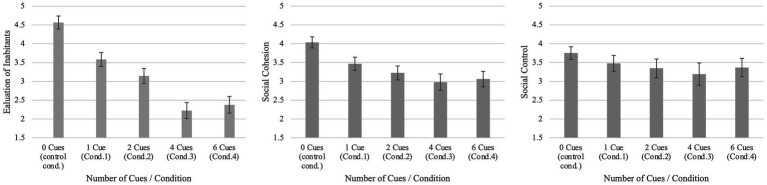
Effects of the condition on the dependent variables. Scales range from 1 to 7; Group means, and 95% CI are depicted.

Detailed analyses are reported in the Supplementary materials S3 for a comprehensive understanding. To examine for the influence of the conditions and the dose dependency on our outcome measures (H4), we conducted separate ANOVAs for each dependent variable and Tukey *Post Hoc* HSD comparisons between the conditions. The means of the conditions were unequal according to a one-way ANOVA for the evaluation of inhabitants *F*(4, 549) = 90.171, *p* < 0.001, *η*^2^ = 0.396, cohesion *F*(4, 549) = 20.967, *p* < 0.001, *η*^2^ = 0.133 and social control *F*(4, 549) = 5.341, *p* < 0.001, *η*^2^ = 0.037. However, the impact of the condition on the dependent variables exhibited variation, with the evaluation of inhabitants demonstrating the strongest effect and the perception of social control displaying the weakest effect. Detailed Tukey *Post Hoc* comparisons between the conditions are reported in Supplementary materials S3. Figure 3 illustrates the effects of the conditions on the three dependent variables.

The results support H4 and indicate that as the number of right-wing graffiti increases, there is a decrease in the perceived qualities of the neighborhoods across all measured constructs. This effect is particularly pronounced in the evaluation of inhabitants compared to cohesion and control. One possible explanation for this pattern is that it is easier for individuals to attribute the perceived information conveyed by political graffiti directly to the people living in the neighborhood (Festinger, 1957). In contrast, assessing cohesion and control might involve understanding the complex dynamics and relationships *between* the inhabitants – information, that might be difficult to assess from our stimuli material. From this perspective, right-wing graffiti would directly result in an intergroup-based perception of neighborhoods (Arceneaux et al., 2013) devaluating those who are presumed to be responsible for creating the political graffiti (Stroud, 2014).

Furthermore, serial comparisons of the means using Tukey’s Honestly Significant Difference (HSD) procedure revealed medium to large effects between the control group and condition 1. For evaluation of inhabitants it was *t* (549) = 6.86, *p* < 0.001, *d* = 0.92; for cohesion it was *t* (549) = 4.31, *p* < 0.001, *d* = 0.58. For control, the effect between the control group and condition 1 was not significant *t* (549) = 2.32, *p* = 0.139, *d* = 0.31. These findings suggest that even a small dose of rightwing graffiti has the potential to influence the perception of neighborhood qualities and their inhabitants. Interestingly, our results indicate a non-linear relationship, as there were no significant differences between conditions 3 and 4 in all dependent variables. This may be due to a saturation effect of the stimulus, right-wing graffiti, which aligns with previous research on this topic (Arendt, 2015). The results further demonstrate that, for individuals with a mean value in political orientation, additional graffiti beyond a critical number does not increase the effect.

To test the interaction hypothesis (H5) we conducted moderation analyses using the PROCESS Macro for R (Hayes, 2022). Social cohesion, evaluation of inhabitants, and social control were set as dependent variables, while condition served as the independent variable, and political orientation (z-transformed) was included as the moderator. Table 3 shows the regression coefficients, R^2^ and *ΔR^2^* for the interaction. To compare each condition to the control group, we utilized simple dummy coding. Consequently, the group means in the conditions when the moderator is at zero can be calculated from the effect sizes of the control condition and condition.

**Table 3:** Regression results for dependent variables.

	Evaluation of inhabitants			Social cohesion			Social control			Exploratory: feeling thermometer foreigners		
Predictor	b	SE	b	b	SE	b	b	SE	b	b	SE	b
			95% CI			95% CI			95% CI			95% CI
			[LL, UL]			[LL, UL]			[LL, UL]			[LL, UL]
Control group	4.56^**^	0.11	[4.38, 4.75]	4.05^**^	0.09	[3.86, 4.22]	3.82^**^	0.11	[3.61, 4.04]	67.21^**^	1.48	[64.30, 70.12]
Group 1: 1 cue	−0.97^**^	0.14	[−1.25, −0.69]	−0.58^**^	0.13	[−0.84, −0.32]	−0.37^*^	0.15	[−0.67, −0.06]	0.65	2.18	[−3.63, 4.92]
Group 2: 2 cues	−1.43^**^	0.14	[−1.70, −1.15]	−0.83^**^	0.13	[−1.09, −0.57]	−0.53^**^	0.16	[−0.84, −0.22]	−3.77	−1.74	[−8.03, −0.49]
Group 3: 4 cues	−2.34^**^	0.14	[−2.62, −2.06]	−1.07^**^	0.13	[−1.34, −0.81]	−0.69^**^	0.16	[−1.01, −037]	3.67	2.21	[−0.67, 8.02]
Group 4: 6 cues	−2.19^**^	0.13	[−2.46, −1.93]	−0.99^**^	0.13	[−1.24, −0.75]	−0.50^**^	0.15	[−0.80, −0.20]	4.12^*^	2.08	[0.05, 8.21]
Political orientation	−0.25^**^	0.09	[−0.43, −0.07]	−0.14	0.09	[−0.31, 0.032]	−0.15	0.10	[−0.35, −0.20]	−11.98^**^	1.42	[−14.75, −9.19]
Group 1 x pol. Or.	0.35^*^	0.14	[0.08, 0.63]	0.15	0.13	[−0.11, 0.42]	0.13	0.16	[−0.19, 0.44]	1.15	2.19	[−3.13, 5.45]
Group 2 x pol. Or.	0.43^**^	0.14	[0.17, 0.70]	0.24	0.13	[−0.01, 0.49]	0.22	0.15	[−0.09, 0.52]	0.38	2.09	[−3.73, 4.49]
Group 3 x pol. Or.	0.39^**^	0.14	[0.11, 0.67]	0.37^**^	0.14	[0.10, 0.63]	0.33^*^	0.16	[0.01, 0.65]	2.47	2.23	[−1.91, 6.85]
Group 4 x pol Or.	0.67^**^	0.13	[0.41, 0.93]	0.52^**^	0.12	[0.27, 0.76]	0.24	0.15	[−0.04, 0.54]	2.25	2.03	[−1.74, 6.23]
Model fit			*R^2^* = 0.43^**^			*R^2^* = 0.17^**^			*R^2^* = 0.05^**^			*R^2^* = 0.33^**^
R2 change interaction			*ΔR^2^* = 0.03^**^			*ΔR^2^* = 0.03^**^			*ΔR^2^* = 0.01			*ΔR^2^* = 0.00

^*^p < 0.05; ^**^p < 0.01.

The model *ΔR^2^* for the interaction between political orientation and condition was significant for the evaluation of inhabitants Δ*R*^2^ = 0.03, *p* < 0.001 and social cohesion Δ*R*^2^ = 0.03, *p* < 0.001, but not for social control Δ*R*^2^ = 0.03, *p* = 0.273. The interactions were significant in all experimental conditions for the dependent variable evaluation of inhabitants. For social cohesion, the interaction only reached levels of significance in groups 3 and 4. For social control, the interaction was only significant in condition 3. The positive regression coefficients of the interaction terms provide evidence that individuals with a right-leaning political orientation tend to evaluate the neighborhoods and their inhabitants more positively compared to those with a left-leaning political orientation. These findings align with our moderation hypothesis and suggest that political orientation moderates the relationship between the conditions and the perception of neighborhoods.

However, it is important to note the moderation hypothesis did not hold across all measured constructs and conditions, which may be attributed to the effect sizes of the conditions.

Additionally, the distribution of political orientation in Study 3 was skewed toward the political left, which could have influenced the results. Considering this skew distribution, even individuals in our sample who represent rightists, our manipulation material may have found our manipulation material which included legally forbidden symbols such as the swastika, too extreme. Despite these considerations, the reported results of the moderation remain highly valuable. Hey indicate that even individuals with a moderate right-leaning political orientation experience less confrontation with the presented stimuli, leading to more favorable ratings of neighborhoods compared to those with a left-leaning political orientation.

In a final step, we conducted an exploratory analysis (E1) to examine the impact of our manipulation onto the attitudes toward foreigners living in Germany. We used a feeling thermometer to measure if the manipulation had the potential to influence more distal attitudes that were not directly related to the neighborhoods but connected to the overall manipulation (e.g., graffiti containing messages like “foreigners out”). However, the results of this exploratory analysis were not conclusive. We found a significant positive effect (*b* = 4.12, [0.05, 8.21]) in condition 4 where all pictures depicting right-wing graffiti, indicating that the group means between the control condition and condition 4 were significantly different. However, we observed no interaction with participants’ political orientation. The finding of a significantly higher group mean in the last condition might be the result of a backlash or resistance hypothesis (Arceneaux et al., 2013), suggesting that participants rejected the implied notion of xenophobia. However, upon closer examination, this explanation weakens on as one would expect an interaction effect, with more leftist participants being bolstered in their initial position (i.e., having more positive attitudes toward foreigners) compared to those oriented toward the right who might agree with the underlying statements. Our data did not indicate such an interaction in the manipulations. These initial results provide an interesting starting point for further studies and highlight the complexity of attitudes toward foreigners in the context of our manipulation.

In conclusion, the results of Study 3 not only replicated but also extended the initial findings from Study 1 regarding the impact of right-wing political graffiti. Through the use of a dose–response approach and precise measurement instruments, we demonstrated that even a small number of right-wing graffiti had a significant influence on the evaluation of neighborhood qualities, particularly the evaluation of its inhabitants. Moreover, we successfully replicated the moderation effect of political orientation, indicating that both attitudinal and counter-attitudinal graffiti affected the evaluation of the neighborhood accordingly.

It is important to acknowledge a limitation of our study, namely the focus exclusively on right-wing graffiti. Due to financial constraints, we were unable to include left-wing graffiti in this study, which would have necessitated a doubling of the sample size. However, we recognize the importance of investigating the effects of left-wing graffiti as well, and we recommend that future research addresses this question to provide a more comprehensive understanding of the topic.

## General discussion

Across three studies, we successfully demonstrated the impact of political graffiti on the perception of previously unfamiliar neighborhoods. Consistent with our hypotheses, this effect was moderated by participants’ political orientation, aligning with existing the literature on political attitudinal and counter-attitudinal messaging (Camaj, 2019), as well as underlying theories of information exposure (Festinger, 1957). Furthermore, our findings indicate that political graffiti is mainly attributed to the inhabitants of a neighborhood. This supports the notion, that intergroup relations play a significant role in the evaluation of neighborhoods, shaping responses as a manifestation of intergroup solidarity or tension.

This ingroup-outgroup dynamic is particularly evident in the results of Study 1, where individuals who identified themselves as leftists displayed similarly high ratings for social cohesion in pictures featuring leftist graffiti as in the control groups. This positive rating can be interpreted as a demonstration of ingroup solidarity. Conversely, the presence of right-wing graffiti resulted in the devaluation of the potential outgroup. Study 1 suggests that this process operates for both political orientations. The results of Study 3 confirm this effect and even extend the findings suggesting a dose–response dependency between the amount of right-wing graffiti and the evaluation of the neighborhoods and their inhabitants.

Further, our results offer compelling evidence to suggest that the principles of the Broken Windows Theory (Kelling and Wilson, 1982) extend beyond traditional indicators of incivilities to include political graffiti representing opposing political views. In this regard, such signs may contribute to a form of politicized segregation or avoidance of certain areas. The implications of this phenomenon are significant, as it implies that individuals may associate neighborhoods with unfavorable qualities, perceiving the inhabitants as unfriendly, dangerous, or holding less favorable neighborhood norms simply based on their presumed opposing political views.

This finding highlights the potential for political ideologies to shape perceptions and attitudes toward neighborhoods, thereby influencing social interactions and community dynamics. The inclusion of political graffiti as a relevant factor in understanding neighborhood evaluations underscores the nuanced ways in which political symbolism can impact individuals’ impressions and behaviors. By illuminating this link between political graffiti and neighborhood perceptions, our studies add a new dimension to the ongoing discourse surrounding the Broken Windows Theory and its implications for social order and community cohesion (Kelling and Wilson, 1982). It underscores the need for further research and policy considerations to address the potential consequences of politicized segregation and its impact on social cohesion within neighborhoods (Motyl et al., 2020).

The results of our experiments also suggest that political graffiti can have significant consequences for residents already living in affected neighborhoods. However, it is important to recognize that this evaluation of norms based on political messaging can have dual effects. On one hand, when political messages align, it may strengthen the social bonds and solidarity among like-minded individuals, reinforcing positive evaluations of neighborhood dynamics. On the other hand, for those holding opposing views, it can lead to lower attachment and decreased trust in the community. Thus, our research adds another layer to the hotly debated topic in the field of community psychology concerning the relationship between diversity and sense of community (Neal, 2017; Craig et al., 2018).

Our studies revealed that participants who encountered political graffiti with messages that did not align with their own political views rated social cohesion and positive neighborhood interactions as less likely. These findings are concerning because positive neighborhood norms, often referred to as the “social glue,” play a vital role in fostering community cohesiveness (Putnam, 2007; Van Assche et al., 2018). In other words, those who disagree with the messages conveyed by the graffiti may perceive their neighbors less favorably and may be less inclined to seek help or support from them and experience less or no sense of community (Jason et al., 2015; Neal, 2017). In turn, these negative perceptions could contribute to intentions to move and ultimately result in political segregation.

Our findings underscore the potential divisive impact of political graffiti on neighborhood dynamics and highlight the need for interventions that promote inclusive and respectful dialogue among residents with diverse political or cultural perspectives. Efforts to foster positive neighborhood norms and strengthen social cohesion can help mitigate the negative consequences of political segregation and promote a sense of belonging for all residents, regardless of their political views.

### Merits, limitations, and avenues for future research

The present series of studies possesses several notable strengths, contributing to a comprehensive and methodologically rigorous investigation of a previously overlooked social phenomenon in social psychological research: political graffiti (Bloch and Phillips, 2020; Alves Diniz and Stafford, 2021). This widespread and historically significant form of expression has received limited attention in the literature until now. The first strength of our research lies in the utilization of reliable measurement scales specifically tailored to assess the impact of political graffiti perception in our experiments. These scales were carefully tested in Study 2 to meet the specific requirements of our studies, ensuring the validity and robustness of our findings.

Another significant merit of our study is the experimental design employed, allowing for the establishment of causal relationships. Study 1 revealed a dual process, demonstrating that alignment with political attitudes results in positive evaluations of neighborhood dynamics and discordance with less positive evaluations. Building upon this initial finding, Study 3 successfully replicated the effect of politicized perception of right-wing graffiti across a comprehensive range of measures, thus confirming its robustness and providing compelling evidence for neighborhood workers, municipalities, and policymakers seeking to foster more positive neighborhood norms.

It is worth emphasizing that political graffiti holds the potential to either harm or enhance neighborhoods and should be approached with caution. Our findings suggest that it could even be utilized as a tool for promoting desirable neighborhood norms, if formulated in the right way (Tankard and Paluck, 2016). These insights are of practical significance and can inform interventions and strategies aimed at creating and maintaining positive community dynamics. By recognizing the influence of political graffiti, stakeholders can better understand its implications and work toward fostering inclusive and cohesive neighborhoods.

While our studies have provided valuable insights into the interactive effects of political attitudes and political graffiti on shaping neighborhood perceptions, they also highlight certain limitations and raise new questions for further research. First and foremost, it is important to acknowledge that graffiti is considered a misdemeanor and as such can be perceived as vandalism or representing a minority opinion. This aspect might diminish the overall effect of the underlying political message as the prevailing attitude might not be perceived as dominant among the neighborhood inhabitants. Consequently, the ability of political graffiti to effectively communicate social norms may be compromised. This limitation could be overcome by exploring alternative forms of political messaging that are perceived as more legitimate and intentional by the community (Hogg and Reid, 2006). For instance, the use of murals, posters, signs displaying voting intentions (as commonly observed in the United States), flags or other symbols carrying political messages could be more strongly associated with formalized representations of the underlying opinions in the neighborhood. This shift toward more official and accepted forms of political expression may enhance the association between political messages and the perception of social norms in neighborhoods.

Furthermore the saturation of official political representations within a neighborhood could provide an even stronger basis for interpreting these cues as indication of a prevailing social norm with greater majority support (Hogg and Reid, 2006; Morris et al., 2015). Ideally, future research should aim to investigate the effects of more official and diverse forms of political messaging on neighborhood perceptions, as well as explore the dose–response dependency of these messages. Again, these studies could help to inform policies and interventions aimed at promoting positive social norms and community cohesion.

Furthermore, it would be valuable to replicate Study 3 by incorporating left-wing graffiti to gain additional insights and we appreciate further research into this direction. If aligning political graffiti can indeed lead to even more positive evaluations of inhabitants compared to the control condition, as suggested by our preliminary findings in Study 1, it would further support the notion that political messages, whether in the form of graffiti or other representations, have the potential to foster a stronger sense of community and cohesion within neighborhoods.

Ideally, future (field) experiments should be conducted to investigate the effects we have presented in real, existing neighborhoods. While self-reported methods have their merits, they also have limitations. Therefore, incorporating other methodological techniques, such as observations of behavior and interactions (Cialdini et al., 1991), would provide a more comprehensive understanding of local neighborhood life. By observing actual behaviors and interactions within the community with the presence of graffiti, we could gain deeper insights into the complex processes involved in community building among inhabitants.

Our findings add to the growing jet, inconclusive discussion of the relationship between various forms of diversity and sense of community (Neal and Neal, 2014; Neal, 2017) by extending the debate on political diversity within communities. Our findings highlight the need to consider diversity on a much broader spectrum when investigating community dynamics. Thus, conducting further studies with diverse neighborhoods would allow for a more comprehensive exploration of the effects of political graffiti on neighborhood perceptions. Different neighborhoods may have unique characteristics and dynamics that could influence the impact of political messaging. Therefore, a broader range of neighborhoods should be included in future research to capture the nuances and variations in the relationship between political graffiti and community perceptions.

Lastly, the preliminary findings of our exploratory analysis on attitudes toward foreigners highlight the need for further investigation on the effects of political graffiti on more distal political attitudes. Specifically, it raises important questions about how the representation of a predominant political attitude shapes intergroup attitudes and intergroup contact toward other groups. This line of inquiry aligns with the Normative Theory in Intergroup Relations proposed by Pettigrew (1991) and builds upon early research conducted in the US, which found that discriminatory behavior of white individuals toward black individuals varied depending on the prevailing social norm in a given social environment (Minard, 1952; Pettigrew, 1958).

To advance our understanding of intergroup relations in everyday life, future research should revive and expand upon the concept of situational and normative influence on socially desired behavior. By investigating the interplay between political attitudes, social norms, and intergroup dynamics, we can gain valuable insights into the mechanisms underlying intergroup relations and potentially identify strategies to promote positive intergroup interactions.

## Conclusion

The current set of experimental studies shed light on the complex interplay between political orientation and the influence of graffiti as a symbolic representation of social norms on neighborhood perceptions. In conclusion, our research serves as an initial step in exploring the theoretical framework of normative influences through cues in the urban environment on intergroup relations in neighborhoods. We believe that our results could help to inform policy makers and community managers to implement better interventions aimed at promoting positive social norms and intergroup harmony within neighborhoods, eventually overcoming the cultural or political divide within society. We hope that our findings will inspire and motivate future research to employ diverse methodological approaches to enhance our understanding of the processes underlying community building and the role of political messaging within neighborhoods.

## Data availability statement

The datasets presented in this study can be found in online repositories. The names of the repository/repositories and accession number(s) can be found at: https://osf.io/2cmwr/.

## Ethics statement

Ethical review and approval was not required for the study on human participants in accordance with the local legislation and institutional requirements. The patients/participants provided their written informed consent to participate in this study.

## Author contributions

CP: planning, designing and running the experiments and studies, preparing and writing the manuscript and other materials. FA: feedback on planned studies, preregistrations, materials, designs, experiments and final documents. All authors contributed to the article and approved the submitted version.

## Funding

The publication of this article was funded by Chemnitz University of Technology and the Deutsche Forschungsgemeinschaft (DFG, German Research Foundation) – Grant number 491193532.

## Conflict of interest

The authors declare that the research was conducted in the absence of any commercial or financial relationships that could be construed as a potential conflict of interest.

## Publisher’s note

All claims expressed in this article are solely those of the authors and do not necessarily represent those of their affiliated organizations, or those of the publisher, the editors and the reviewers. Any product that may be evaluated in this article, or claim that may be made by its manufacturer, is not guaranteed or endorsed by the publisher.

## Footnotes

^1^We preregistered two exploratory analyses for Study 2 that use repeated measure ANOVA to test perceived acculturation attitudes of the inhabitants in the presented neighborhoods. For transparency reasons, we report the analyses in the Supplementary materials. The Analysis however did not impact the results of Study 2 or decisions made for Study 3.
