# Supplementary material for: If graffiti changed anything, it would be illegal. The influence of political graffiti on the perception of neighborhoods and intergroup attitudes
Source: Front Psychol. 2023 Jul 13;14:1098105. doi: 10.3389/fpsyg.2023.1098105 (PMC10403061; doi:10.3389/fpsyg.2023.1098105)
Supplement: Supplementary file 1 [file Data_Sheet_1.docx]

**Supplemental Materials (S1) for: If Graffiti Changed Anything, It Would Be Illegal. The Influence of Political Graffiti on the Perception of Neighborhoods and Intergroup Attitudes.**

**Study 1**

**Overview:** For each triplet of pictures, we report:

1. the used material
2. the structure of the dependent variable using exploratory factor analysis.
3. scale reliabilities using Cronbach’s alpha.
4. results of the manipulation check
5. analysis, results, and plotted data of the interaction

**Table of Content**

[Materials for Picture 1 2](#_Toc109641653)

[Materials for Picture 2 5](#_Toc109641654)

[Materials for Picture 3 8](#_Toc109641655)

[Materials for Picture 4 11](#_Toc109641656)

[Materials for Picture 5 15](#_Toc109641657)

[Materials for Picture 6 18](#_Toc109641658)

[Materials for Picture 7 21](#_Toc109641659)

[Materials for Picture 8 24](#_Toc109641660)

[Materials for Picture 9 27](#_Toc109641661)

# Materials for Picture 1

| Neutral Condition | Left-wing Condition | Right-wing Condition |
| --- | --- | --- |
| 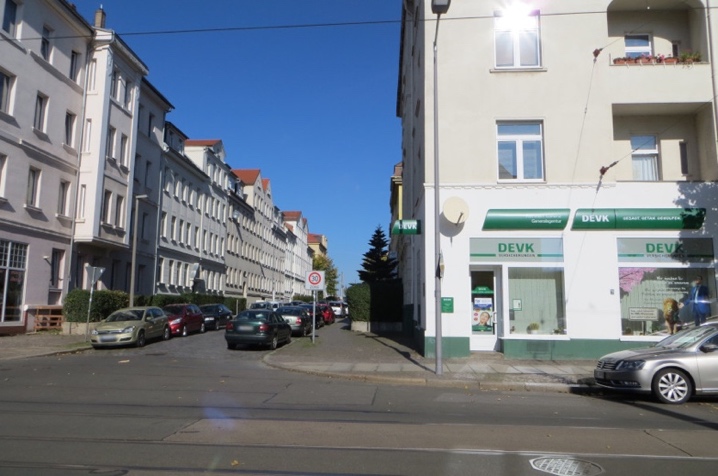 | 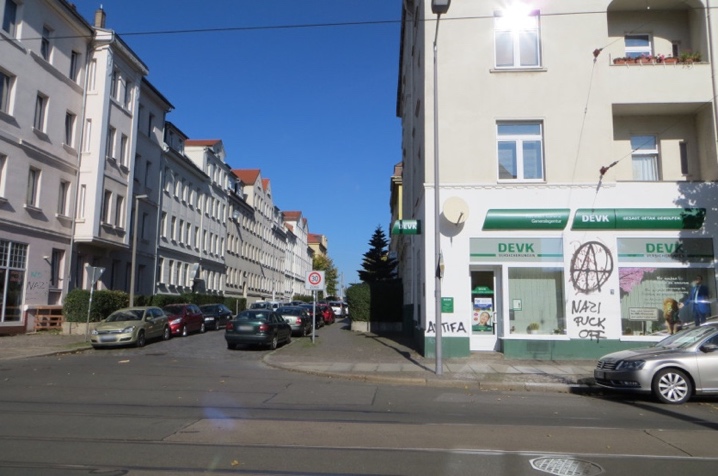 | 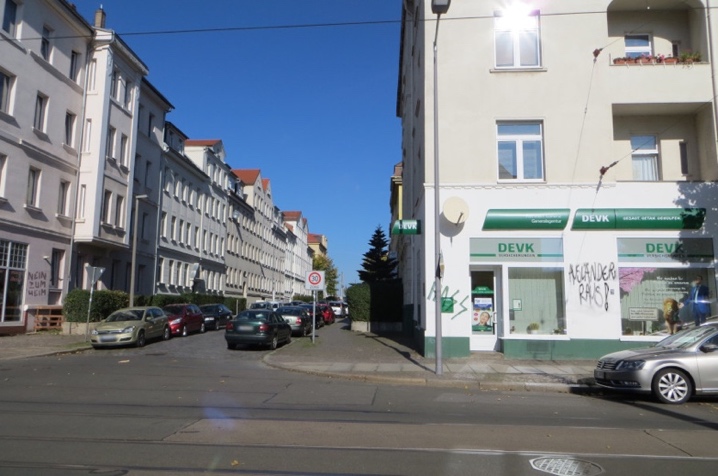 |

**Analysis of Depended Variable Cohesion in Picture 1**

An exploratory factor analysis (EFA) of the six items for social cohesion with oblique rotation (oblimin) revealed a single factor solution with an eigenvalue over 1, (eigenvalue progression = 3.87, 0.79, 0.47) explaining 58% of the total variance. The Kaiser–Meyer–Olkin measure verified the sampling adequacy for the analysis, KMO = .87. All KMO values for individual items were > .84, which is well above the acceptable limit of .5 (Field, 2009). Bartlett’s test of sphericity χ2 (15) = 675.999, *p* < .001, indicated that correlations between items were sufficiently large for EFA.

Cronbach’s Alpha of the scale was .89.

**Manipulation Check for Picture 1**

An analysis of variance (ANOVA) with condition as independent variables and the control item (“what do you think is the political orientation of people living in this neighborhood?”) as dependent variable yielded significant variation among conditions, *F* (2, 196) =11.86, p < .001. A post hoc Tukey test showed that the conditions right-left and right-neutral differed significantly at *p* < .05; left-neutral differed at *p* = .0571.

**Analysis of Interaction in Picture 1**

**Table S1.1**: *Regression results using Cohesion of Picture 1 as the criterion*

| Predictor | *b* | 95% CI  [LL, UL] | Fit |
| --- | --- | --- | --- |
| (Intercept) | 6.35** | [6.13, 6.57] |  |
| Neutral vs. Left+Right | 1.27** | [0.80, 1.73] |  |
| Left vs. Right | 0.21 | [-0.32, 0.74] |  |
| Political Orientation | -0.06 | [-0.28, 0.17] |  |
| Pol. Or. X Neutral vs. Left Right | -0.00 | [-0.43, 0.43] |  |
| Pol. Or. X Left vs Right | -0.30 | [-0.90, 0.30] |  |
|  |  |  | *R^2^*  = .140** |
|  |  |  | 95% CI[.05,.21] |
|  |  |  |  |

*Note.* Linerar Regression using helmert coding. * indicates p < .05. ** indicates p < .01. The moderator political orientation was z-transformed before the anaylsis.

**Results Analysis Picture 1**

We used a helmert coding to calculate the linear regression testing our hypothesis. The regression analysis showed a non-significant interaction, *p* = .29, between political orientation and the left right conditions. A plot of the interaction (*Figure 1*) however revealed the expected pattern.

**Figure S1.1**: Plot of the Interaction in Picture 1


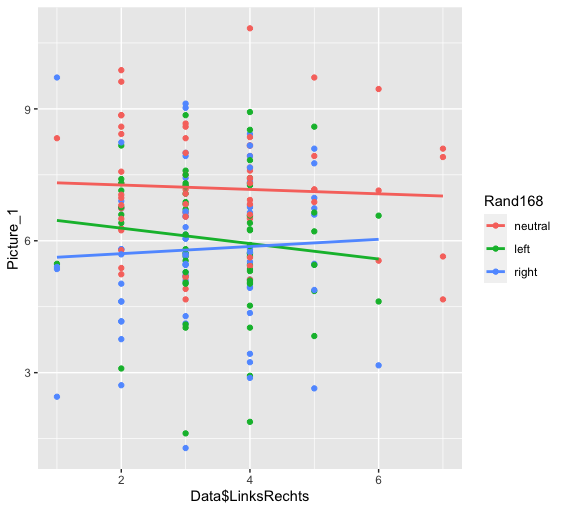


# Materials for Picture 2

| Neutral Condition | Left-wing Condition | Right-wing Condition |
| --- | --- | --- |
| 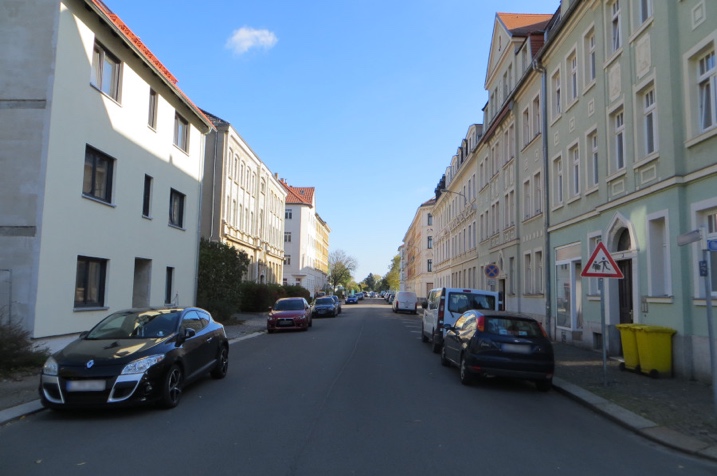 | 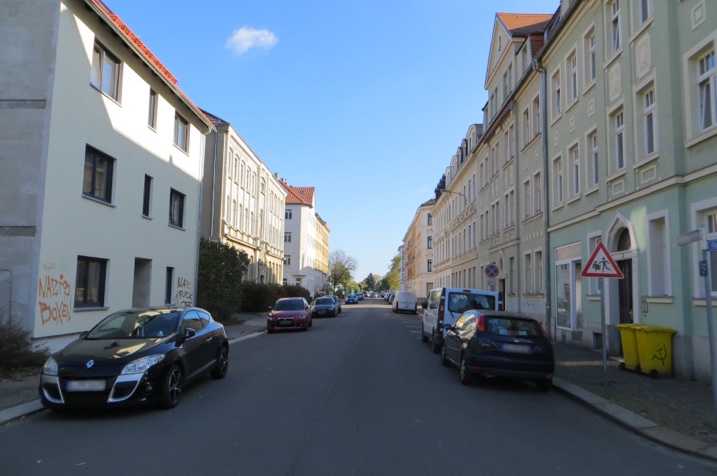 | 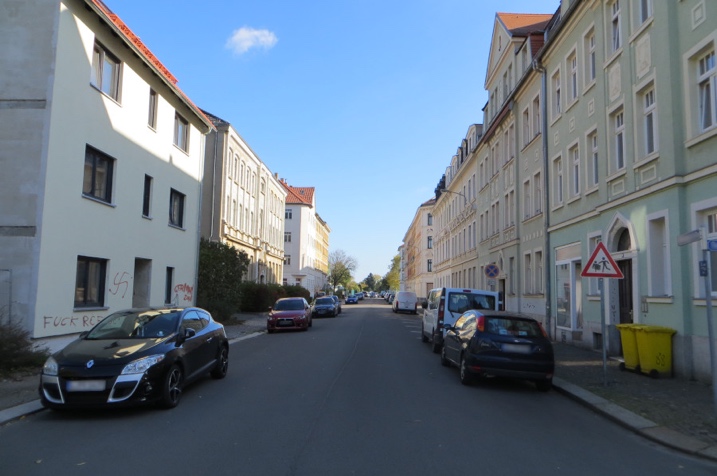 |

**Analysis of Depended Variable Cohesion in Picture 2**

An exploratory factor analysis (EFA) of the six items for social cohesion with oblique rotation (oblimin) revealed a single factor solution with an eigenvalue over 1, (eigenvalue progression = 3.96, 0.73, 0.50) explaining 60% of the total variance. The Kaiser–Meyer–Olkin measure verified the sampling adequacy for the analysis, KMO = .87. All KMO values for individual items were > .84, which is well above the acceptable limit of .5 (Field, 2009). Bartlett’s test of sphericity χ2 (15) = 707.94, *p* < .001, indicated that correlations between items were sufficiently large for EFA.

Cronbach’s Alpha of the scale was .90.

**Manipulation Check for Picture 2**

An analysis of variance (ANOVA) with condition as independent variables and the control item (“what do you think is the political orientation of people living in this neighborhood?”) as dependent variable yielded significant variation among conditions, *F* (2, 196) =32.01, p < .001. A post hoc Tukey test showed that the conditions right-left, right-neutral and left-neutral differed significantly at *p* < .001.

**Analysis of Interaction in Picture 2**

**Table S1.2:** *Regression results using Cohesion of Picture 2 as the criterion*

| Predictor | *b* | 95% CI  [LL, UL] | Fit |
| --- | --- | --- | --- |
| (Intercept) | 6.23** | [6.02, 6.44] |  |
| Neutral vs. Left+Right | 1.50** | [1.05, 1.95] |  |
| Left vs. Right | 0.21 | [-0.30, 0.72] |  |
| Political Orientation | -0.12 | [-0.33, 0.10] |  |
| Pol. Or. X Neutral vs. Left Right | 0.35 | [-0.15, 0.85] |  |
| Pol. Or. X Left vs Right | -0.14 | [-0.61, 0.34] |  |
|  |  |  | *R^2^*  = .200** |
|  |  |  | 95% CI[.09,.28] |
|  |  |  |  |

*Note.* Linerar Regression using helmert coding. * indicates p < .05. ** indicates p < .01.

**Results Analysis 2**

We used a helmert coding to calculate the linear regression testing our hypothesis. The regression analysis showed no significant interaction between political orientation and the left-right conditions. A plot of the interaction (*Figure 2)* showed not the expected pattern.

**Figure S1.2**: Plot of the Interaction for Picture 2


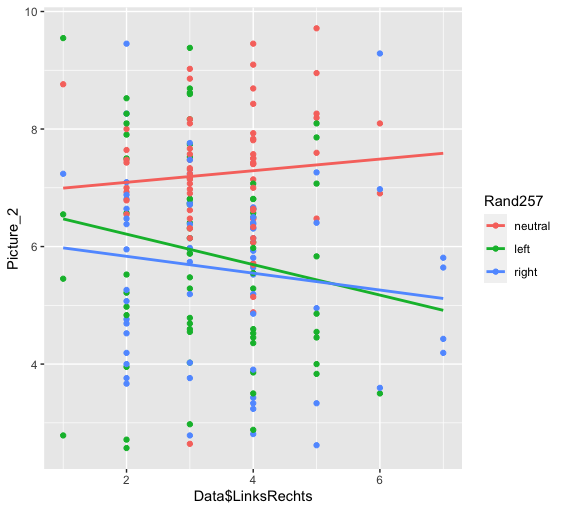


# Materials for Picture 3

| Neutral Condition | Left-wing Condition | Right-wing Condition |
| --- | --- | --- |
| 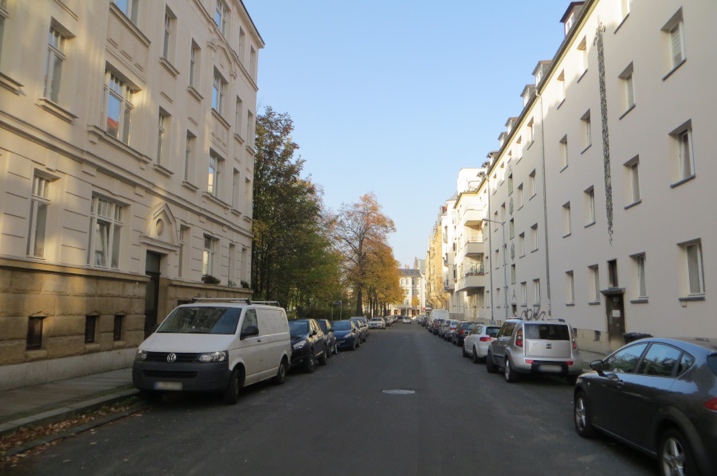 | 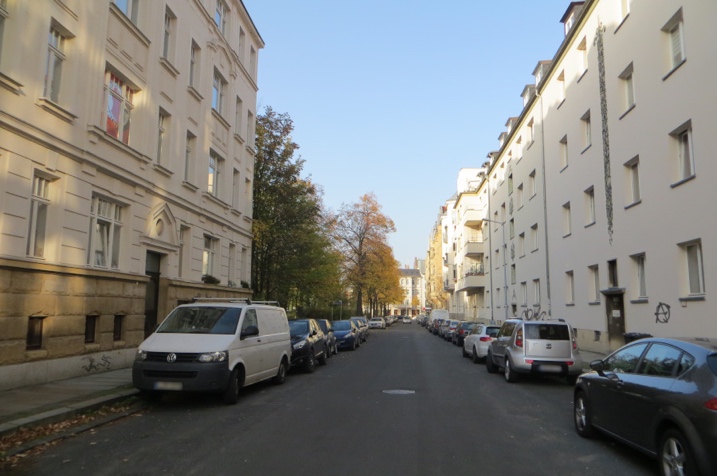 | 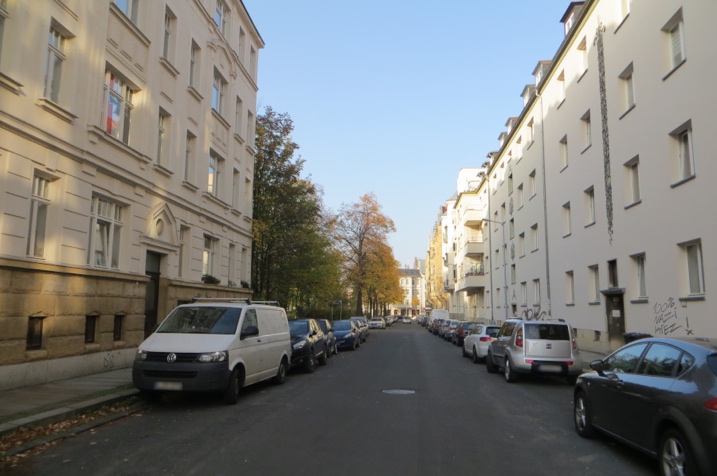 |

**Analysis of Depended Variable Cohesion in Picture 3**

An exploratory factor analysis (EFA) of the six items for social cohesion with oblique rotation (oblimin) revealed a single factor solution with an eigenvalue over 1, (eigenvalue progression = 3.90, 0.84, 0.47…) explaining 59% of the total variance. The Kaiser–Meyer–Olkin measure verified the sampling adequacy for the analysis, KMO = .86. All KMO values for individual items were > .82, which is well above the acceptable limit of .5 (Field, 2009). Bartlett’s test of sphericity χ2 (15) = 723.867, *p* < .001, indicated that correlations between items were sufficiently large for EFA.

Cronbach’s Alpha of the scale was .89.

**Manipulation Check for Picture 3**

An analysis of variance (ANOVA) with condition as independent variables and the control item (“what do you think is the political orientation of people living in this neighborhood?”) as dependent variable yielded significant variation among conditions, *F* (2, 196) =4.06, p < .05. A post hoc Tukey test showed that the condition left-neutral did not differ significantly; left-right and right-neutral differed at *p* < .05.

**Analysis of Interaction in Picture 3**

Table 3: *Regression results using Cohesion of Picture 3 as the criterion*

| Predictor | *b* | 95% CI  [LL, UL] | Fit |
| --- | --- | --- | --- |
| (Intercept) | 6.24** | [6.03, 6.46] |  |
| Neutral vs. Left+Right | 0.76** | [0.30, 1.21] |  |
| Left vs. Right | 0.26 | [-0.28, 0.80] |  |
| Political Orientation | -0.07 | [-0.29, 0.15] |  |
| Pol. Or. X Neutral vs. Left Right | 0.05 | [-0.42, 0.52] |  |
| Pol. Or. X Left vs Right | -0.08 | [-0.63, 0.47] |  |
|  |  |  | *R^2^*  = .063* |
|  |  |  | 95% CI[.00,.12] |
|  |  |  |  |

*Note.* Linerar Regression using helmert coding. * indicates p < .05. ** indicates p < .01.

**Results Analysis 3**

We used a helmert coding to calculate the linear regression testing our hypothesis. The regression analysis showed a non-significant interaction, *p* = .83, between political orientation and the left right conditions. Explained variance of the model was 5.9%. A plot of the interaction (*Figure 1*) revealed not the expected pattern.

**Figure S1.3**: Plotting of the Interaction in Picutre 3

**
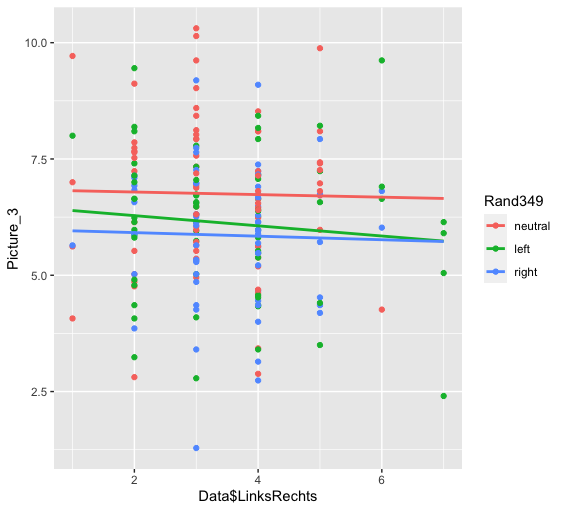
**

# Materials for Picture 4

| Neutral Condition | Left-wing Condition | Right-wing Condition |
| --- | --- | --- |
| 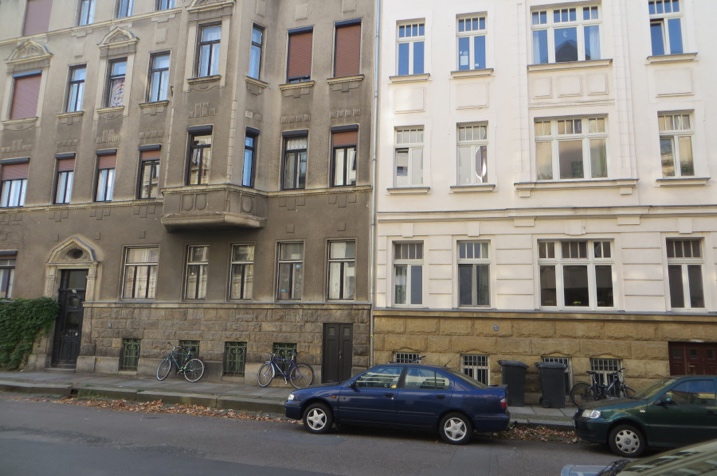 | 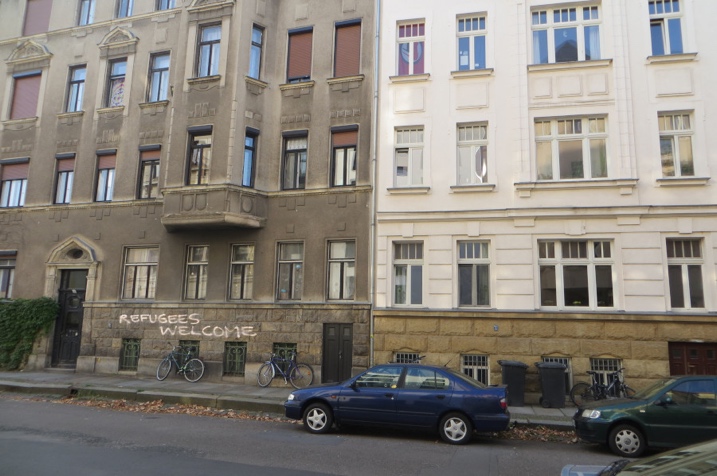 | 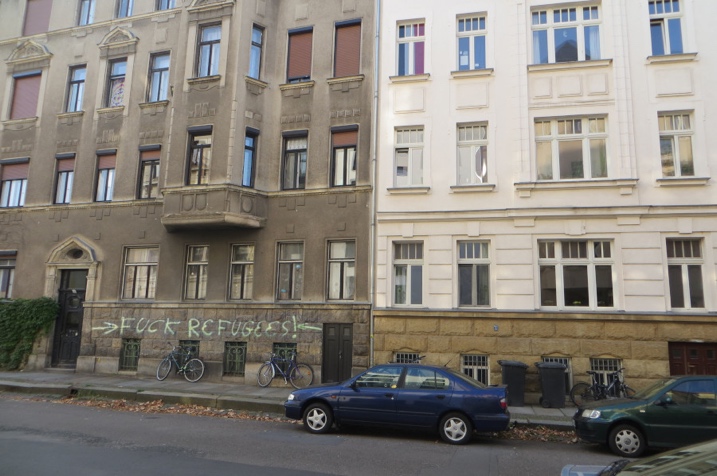 |

**Analysis of Depended Variable Cohesion in Picture 4**

An exploratory factor analysis (EFA) of the six items for social cohesion with oblique rotation (oblimin) revealed a single factor solution with an eigenvalue over 1, (eigenvalue progression = 4.06, 0.78, 0.44…) explaining 62% of the total variance. The Kaiser–Meyer–Olkin measure verified the sampling adequacy for the analysis, KMO = .84. All KMO values for individual items were > .81, which is well above the acceptable limit of .5 (Field, 2009). Bartlett’s test of sphericity χ2 (15) = 790.009, *p* < .001, indicated that correlations between items were sufficiently large for EFA.

Cronbach’s alpha of the scale was .90.

**Manipulation Check for Picture 4**

An analysis of variance (ANOVA) with condition as independent variables and the control item (“what do you think is the political orientation of people living in this neighborhood?”) as dependent variable yielded significant variation among conditions, *F* (2, 196) =119.2, p < .001. A post hoc Tukey test showed that all conditions differed significantly at *p* < .001.

**Analysis of Interaction in Picture 4**

Table 4: *Regression results using Cohesion of Picture 4 as the criterion*

| Predictor | *b* | 95% CI  [LL, UL] | Fit |
| --- | --- | --- | --- |
| (Intercept) | 5.79** | [5.58, 6.00] |  |
| Neutral vs. Left+Right | 0.84** | [0.39, 1.29] |  |
| Left vs. Right | 1.83** | [1.29, 2.36] |  |
| Political Orientation | -0.32** | [-0.53, -0.10] |  |
| Pol. Or. X Neutral vs. Left+Right | 0.02 | [-0.44, 0.48] |  |
| Pol. Or. X Left vs. Right | -0.36 | [-0.90, 0.18] |  |
|  |  |  | *R^2^*  = .280** |
|  |  |  | 95% CI[.16,.36] |
|  |  |  |  |

*Note.* Linerar Regression using helmert coding. * indicates p < .05. ** indicates p < .01.

**Results Analysis 4**

We used a helmert coding to calculate the linear regression testing our hypothesis. The regression analysis showed a non-significant interaction between political orientation and the left right conditions. In this picture, political orientation had a negative influence on the evaluation of the neighborhood. This could be due to the contrast between the two houses and people on the rightwing spectrum prefer an orderly/clean neighborhood. A plot of the model (*Figure 4*) shows a negative slope in the neutral condition.

**Figure S1.4**: Plotting of the Interaction in Picture 4


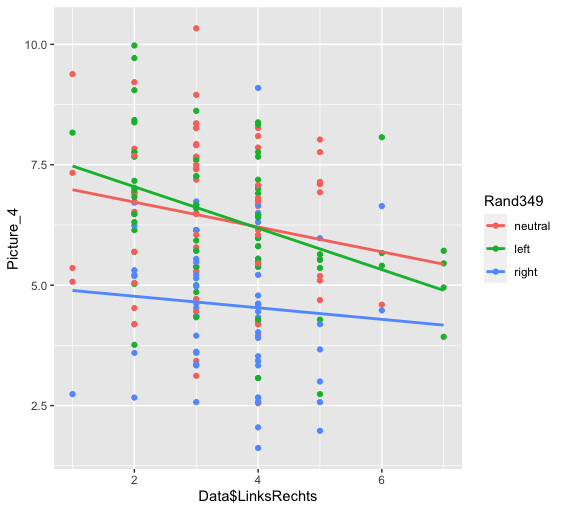


# Materials for Picture 5

| Neutral Condition | Left-wing Condition | Right-wing Condition |
| --- | --- | --- |
| 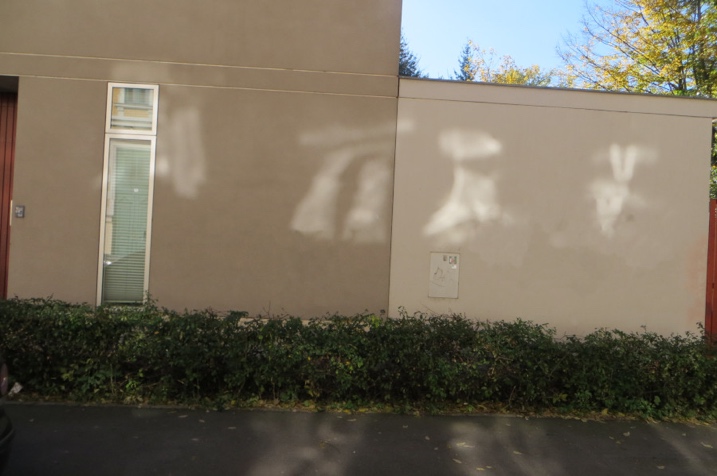 | 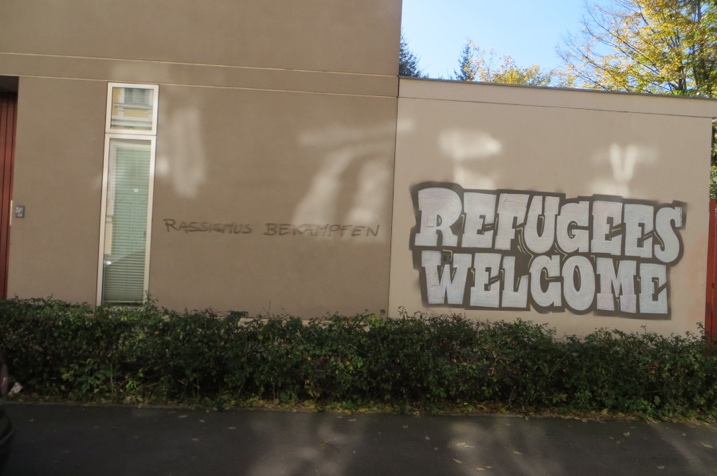 | 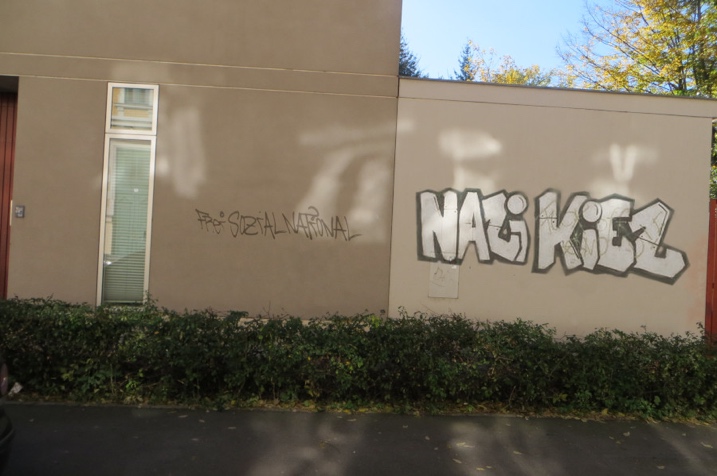 |

**Analysis of Depended Variable Cohesion in Picture 5**

An exploratory factor analysis (EFA) of the six items for social cohesion with oblique rotation (oblimin) revealed a single factor solution with an eigenvalue over 1, (eigenvalue progression = 4.31, 0.70, 0.37…) explaining 67% of the total variance. The Kaiser–Meyer–Olkin measure verified the sampling adequacy for the analysis, KMO = .87. All KMO values for individual items were > .81, which is well above the acceptable limit of .5 (Field, 2009). Bartlett’s test of sphericity χ2 (15) = 919.611, *p* < .001, indicated that correlations between items were sufficiently large for EFA.

Cronbach’s Alpha of the scale was .92.

**Manipulation Check for Picture 5**

An analysis of variance (ANOVA) with condition as independent variables and the control item (“what do you think is the political orientation of people living in this neighborhood?”) as dependent variable yielded significant variation among conditions, *F* (2, 196) =171.70, p < .001. A post hoc Tukey test showed that all conditions differed significantly at *p* < .001.

**Analysis of Interaction in Picture 5**

Table S1.5: *Regression results using Cohesion of Picture 5 as the criterion*

| Predictor | *b* | 95% CI  [LL, UL] | Fit |
| --- | --- | --- | --- |
| (Intercept) | 5.27** | [5.02, 5.53] |  |
| Neutral vs. Left+Right | 0.62* | [0.08, 1.16] |  |
| Left vs. Right | 1.56** | [0.94, 2.17] |  |
| Political Orientation | 0.04 | [-0.22, 0.30] |  |
| Pol. Or. X Neutral vs. Left Right | 0.27 | [-0.33, 0.86] |  |
| Pol. Or. X Left vs Right | -0.68* | [-1.25, -0.11] |  |
|  |  |  | *R^2^*  = .162** |
|  |  |  | 95% CI[.06,.24] |
|  |  |  |  |

*Note.* Linerar Regression using helmert coding. * indicates p < .05. ** indicates p < .01. The moderator Political Orientation was standardized and z-transformed

**Results Analysis Picture 5**

We used a helmert coding to calculate the linear regression testing our hypothesis. The regression analysis showed a significant interaction between political orientation and the left right conditions. A plot of the interaction (*Figure 1*) revealed the expected pattern.

Figure S1.5: Plotting of the Interaction for Picture 5


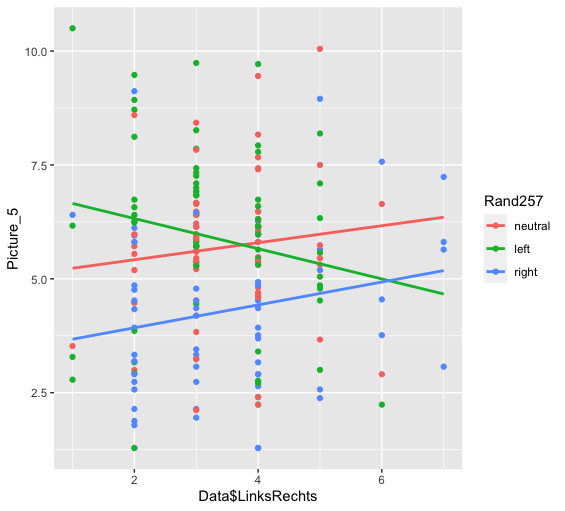


# Materials for Picture 6

| Neutral Condition | Left-wing Condition | Right-wing Condition |
| --- | --- | --- |
| 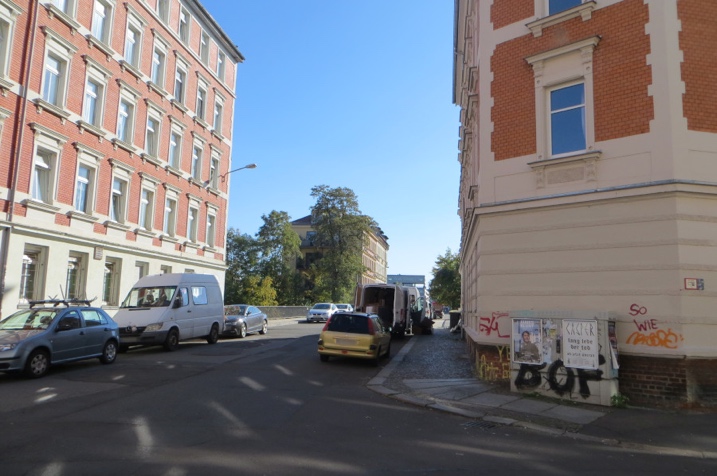 | 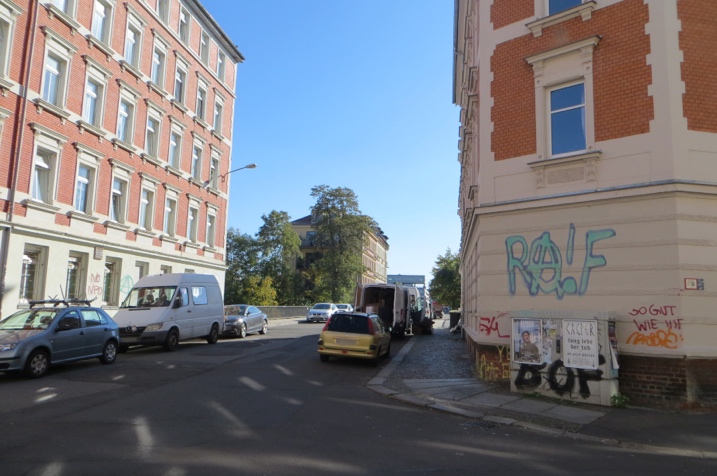 | 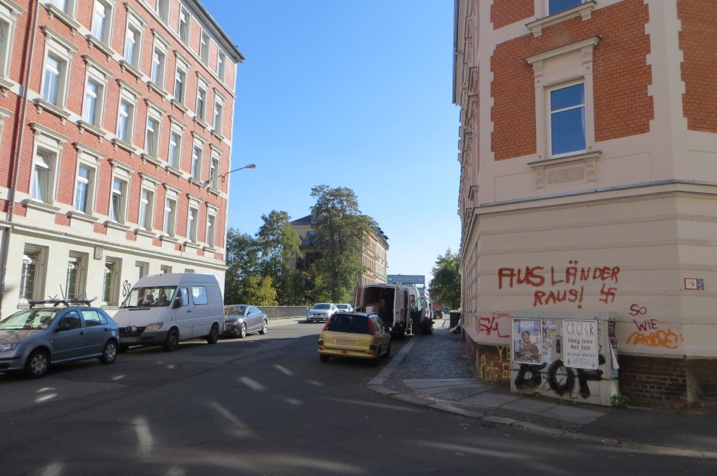 |

**Analysis of Depended Variable Cohesion in Picture 6**

An exploratory factor analysis (EFA) of the six items for social cohesion with oblique rotation (oblimin) revealed a single factor solution with an eigenvalue over 1, (eigenvalue progression = 4.30, 0.63, 0.41…) explaining 66% of the total variance. The Kaiser–Meyer–Olkin measure verified the sampling adequacy for the analysis, KMO = .88. All KMO values for individual items were > .85, which is well above the acceptable limit of .5 (Field, 2009). Bartlett’s test of sphericity χ2 (15) = 886.32, *p* < .001, indicated that correlations between items were sufficiently large for EFA.

Cronbach’s Alpha of the scale was .92.

**Manipulation Check for Picture 6**

An analysis of variance (ANOVA) with condition as independent variables and the control item (“what do you think is the political orientation of people living in this neighborhood?”) as dependent variable yielded significant variation among conditions, *F* (2, 196) =80.72, p < .001. A post hoc Tukey test showed that the conditions right-left and right-neutral differed significantly at *p* < .05; left-neutral did not differ significantly *p* = .170.

**Analysis of Interaction in Picture 6**

**Table** S1.6: *Regression results using Cohesion of Picture 6 as the criterion*

| Predictor | *b* | 95% CI  [LL, UL] | Fit |
| --- | --- | --- | --- |
| (Intercept) | 5.43** | [5.21, 5.65] |  |
| Neutral vs. Left+Right | 0.97** | [0.50, 1.45] |  |
| Left vs. Right | 1.17** | [0.63, 1.71] |  |
| Political Orientation | 0.00 | [-0.22, 0.23] |  |
| Pol. Or. X Neutral vs. Left Right | -0.01 | [-0.45, 0.43] |  |
| Pol. Or. X Left vs Right | -0.53^+^ | [-1.13, 0.08] |  |
|  |  |  | *R^2^*  = .176** |
|  |  |  | 95% CI[.07,.25] |
|  |  |  |  |

*Note.* Linerar Regression using helmert coding. + indicates p < .1* indicates p < .05. ** indicates p < .01.

**Results Analysis Picture 6**

We used a helmert coding to calculate the linear regression testing our hypothesis. The regression analysis showed an close to significant interaction, *p* = .07 between political orientation and the left right conditions. A plot of the interaction (*Figure 1*) however revealed the expected pattern.

**Figure S1.6**: Plotting of the Interaction in Picture 6


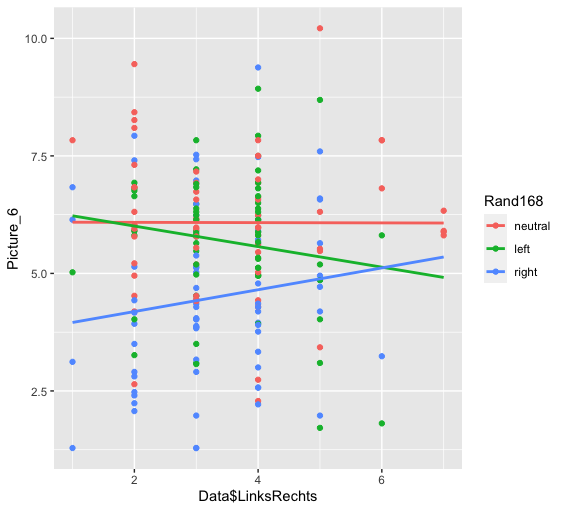


# Materials for Picture 7

| Neutral Condition | Left-wing Condition | Right-wing Condition |
| --- | --- | --- |
| 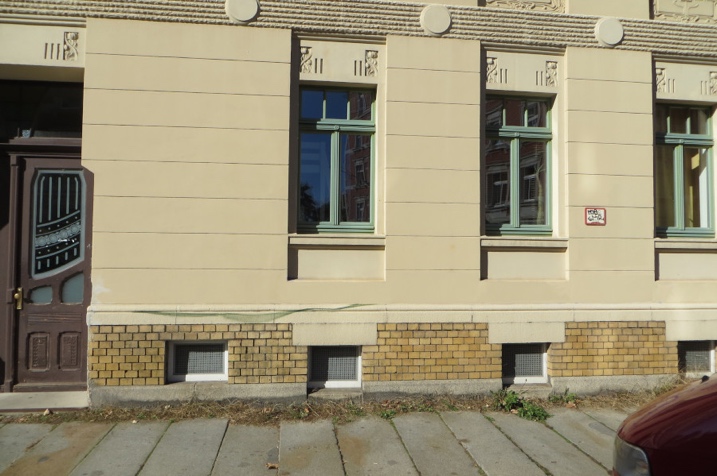 | 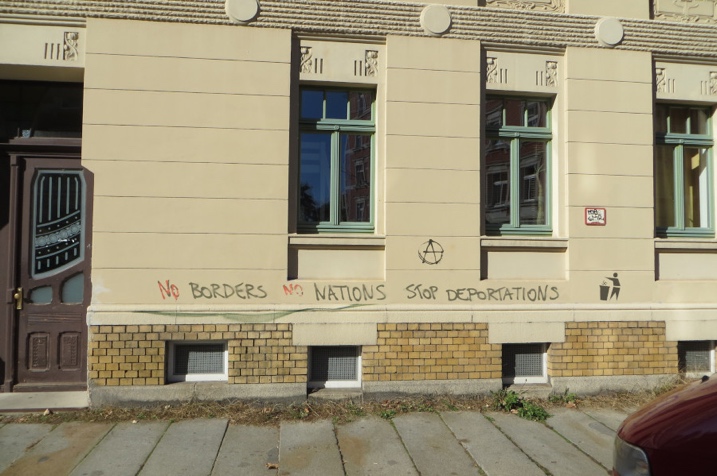 | 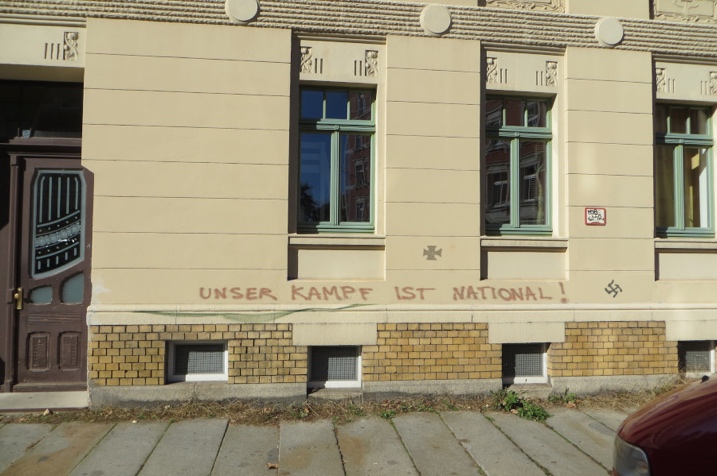 |

**Analysis of Depended Variable Cohesion in Picture 7**

An exploratory factor analysis (EFA) of the six items for social cohesion with oblique rotation (oblimin) revealed a single factor solution with an eigenvalue over 1, (eigenvalue progression = 4.30, 0.74, 0.39…) explaining 67% of the total variance. The Kaiser–Meyer–Olkin measure verified the sampling adequacy for the analysis, KMO = .86. All KMO values for individual items were > .81, which is well above the acceptable limit of .5 (Field, 2009). Bartlett’s test of sphericity χ2 (15) = 953.212, *p* < .001, indicated that correlations between items were sufficiently large for EFA.

Cronbach’s Alpha of the scale was .92.

**Manipulation Check for Picture 7**

An analysis of variance (ANOVA) with condition as independent variables and the control item (“what do you think is the political orientation of people living in this neighborhood?”) as dependent variable yielded significant variation among conditions, *F* (2, 196) =111.1, p < .001. A post hoc Tukey test showed that all conditions differed significantly at *p* < .001.

**Analysis of Interaction in Picture 7**

**Table S1.7**: *Regression results using Cohesion of Picture 7 as the criterion*

| Predictor | *b* | 95% CI  [LL, UL] | Fit |
| --- | --- | --- | --- |
| (Intercept) | 5.39** | [5.17, 5.62] |  |
| Neutral vs. Left+Right | 1.43** | [0.94, 1.91] |  |
| Left vs. Right | 1.01** | [0.46, 1.56] |  |
| Political Orientation | -0.05 | [-0.28, 0.18] |  |
| Pol. Or. X Neutral vs. Left Right | 0.15 | [-0.39, 0.68] |  |
| Pol. Or. X Left vs Right | -0.99** | [-1.50, -0.48] |  |
|  |  |  | *R^2^*  = .237** |
|  |  |  | 95% CI[.12,.32] |
|  |  |  |  |

*Note.* Linerar Regression using helmert coding. * indicates p < .05. ** indicates p < .01.

**Results Analysis Picture 7**

We used a helmert coding to calculate the linear regression testing our hypothesis. The regression analysis showed a significant interaction between political orientation and the left right conditions. Explained variance of the model was 23.7%. A plot of the interaction (*Figure 7*) revealed the expected pattern.

**Figure S1.7**: Plotting of the Interaction in Picture 7


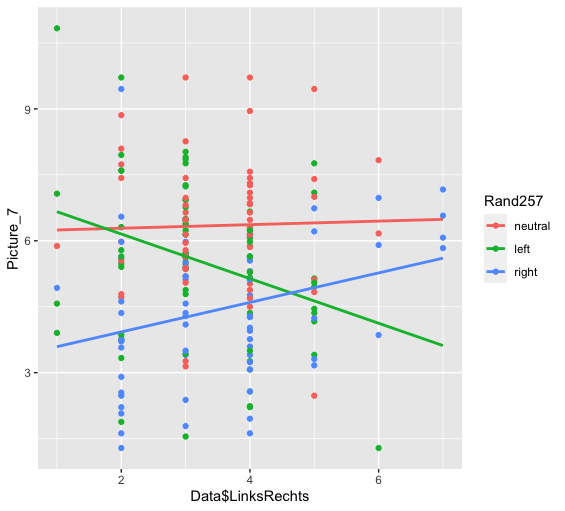


# Materials for Picture 8

| Neutral Condition | Left-wing Condition | Right-wing Condition |
| --- | --- | --- |
| 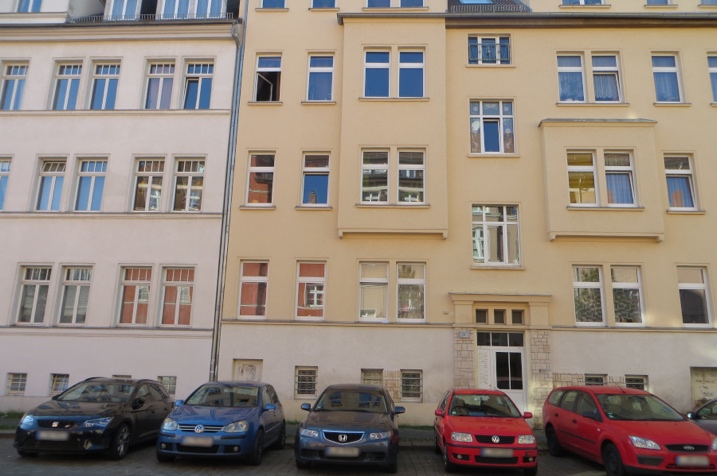 | 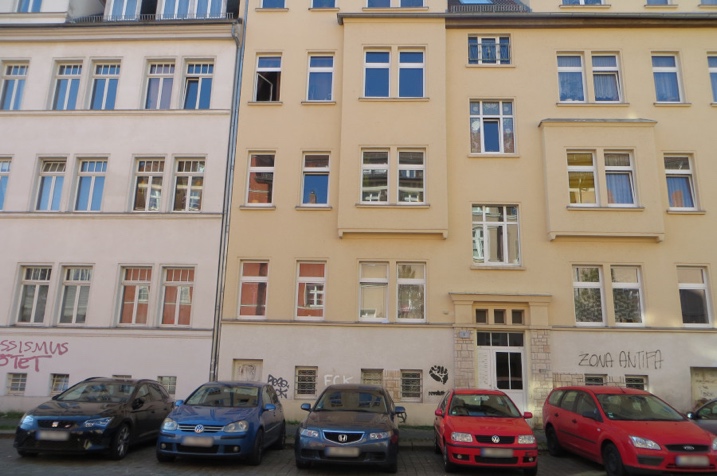 | 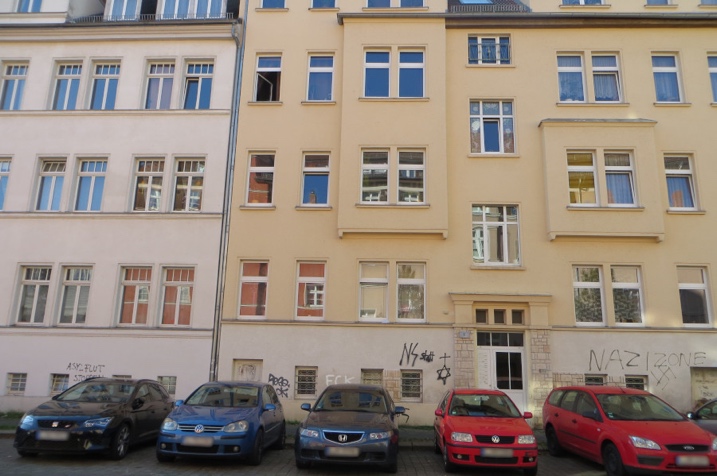 |

**Analysis of Depended Variable Cohesion in Picture 8**

An exploratory factor analysis (EFA) of the six items for social cohesion with oblique rotation (oblimin) revealed a single factor solution with an eigenvalue over 1, (eigenvalue progression = 4.57, 0.56, 0.33…) explaining 62% of the total variance. The Kaiser–Meyer–Olkin measure verified the sampling adequacy for the analysis, KMO = .89. All KMO values for individual items were > .87, which is well above the acceptable limit of .5 (Field, 2009). Bartlett’s test of sphericity χ2 (15) = 1062.039, *p* < .001, indicated that correlations between items were sufficiently large for EFA.

Cronbach’s Alpha of the scale was .94.

**Manipulation Check for Picture 8**

An analysis of variance (ANOVA) with condition as independent variables and the control item (“what do you think is the political orientation of people living in this neighborhood?”) as dependent variable yielded significant variation among conditions, *F* (2, 196) =77.45, p < .001. A post hoc Tukey test showed that all conditions differed significantly at *p* < .01.

**Analysis of Interaction in Picture 8**

Table 8: *Regression results using Cohesion of Picture 8 as the criterion*

| Predictor | *b* | 95% CI  [LL, UL] | Fit |
| --- | --- | --- | --- |
| (Intercept) | 5.35** | [5.12, 5.58] |  |
| Neutral vs. Left+Right | 2.29** | [1.80, 2.78] |  |
| Left vs. Right | 1.38** | [0.82, 1.93] |  |
| Political Orientation | -0.05 | [-0.28, 0.19] |  |
| Pol. Or. X Neutral vs. Left Right | 0.07 | [-0.38, 0.52] |  |
| Pol. Or. X Left vs Right | -0.53^+^ | [-1.15, 0.10] |  |
|  |  |  | *R^2^*  = .380** |
|  |  |  | 95% CI[.26,.46] |
|  |  |  |  |

*Note.* Linerar Regression using helmert coding. ^+^ indicating p < .1 * indicates p < .05. ** indicates p < .01.

**Results Picture 8**

We used a helmert coding to calculate the linear regression testing our hypothesis. The regression analysis showed an interaction between political orientation and the left right conditions with *p* = .08. Explained variance of the model was 38%. A plot of the interaction (*Figure 1*) revealed the expected pattern.

**Figure S1.8**: Plotting of the Interaction

**
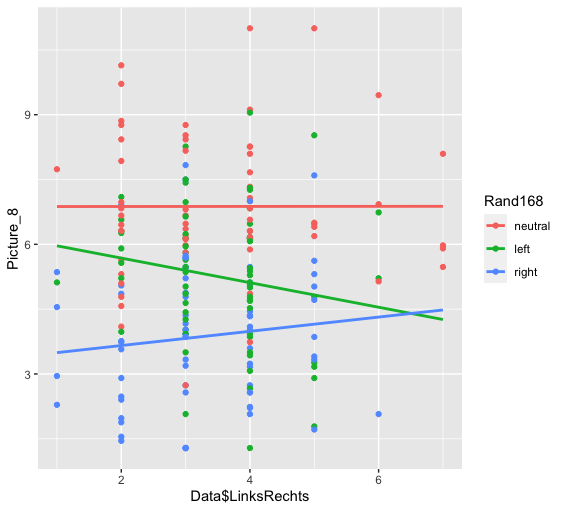
**

# Materials for Picture 9

| Neutral Condition | Left-wing Condition | Right-wing Condition |
| --- | --- | --- |
| 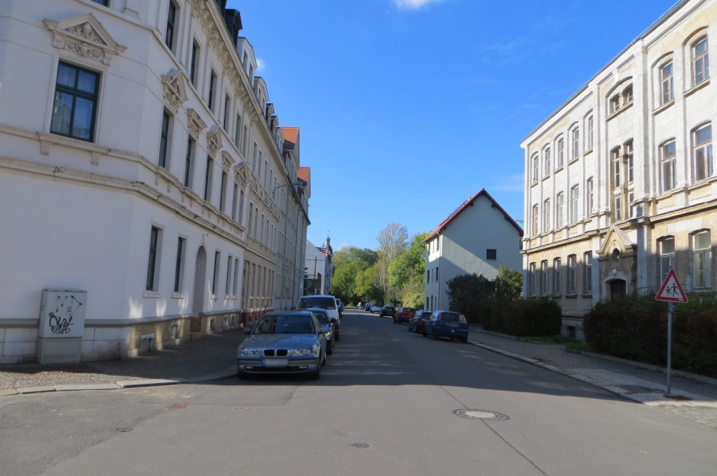 | 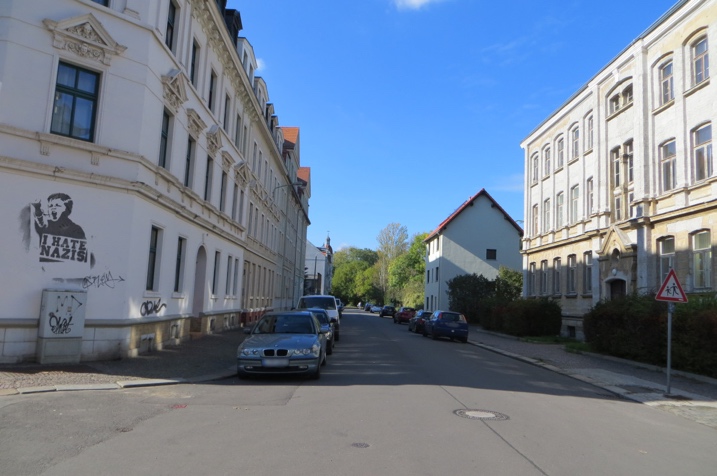 | 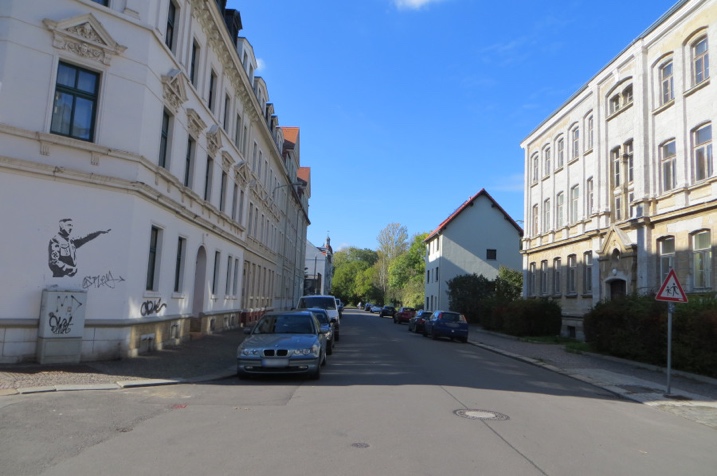 |

**Analysis of Depended Variable Cohesion in Picture 9**

An exploratory factor analysis (EFA) of the six items for social cohesion with oblique rotation (oblimin) revealed a single factor solution with an eigenvalue over 1, (eigenvalue progression = 4.26, 0.64, 0.46) explaining 66% of the total variance. The Kaiser–Meyer–Olkin measure verified the sampling adequacy for the analysis, KMO = .88. All KMO values for individual items were > .83, which is well above the acceptable limit of .5 (Field, 2009). Bartlett’s test of sphericity χ2 (15) = 893.656, *p* < .001, indicated that correlations between items were sufficiently large for EFA.

Cronbach’s Alpha of the scale was .92.

**Manipulation Check for Picture 9**

An analysis of variance (ANOVA) with condition as independent variables and the control item (“what do you think is the political orientation of people living in this neighborhood?”) as dependent variable yielded significant variation among conditions, *F* (2, 196) =95.48, p < .001. A post hoc Tukey test showed that all conditions differed significantly at *p* < .001.

**Analysis of Interaction in Picture 9**

Table S1.9: *Regression results using Cohesion of Picture 1 as the criterion*

| Predictor | *b* | 95% CI  [LL, UL] | Fit |
| --- | --- | --- | --- |
| (Intercept) | 6.17** | [5.95, 6.39] |  |
| Neutral vs. Left+Right | 1.75** | [1.29, 2.20] |  |
| Left vs. Right | 1.17** | [0.63, 1.71] |  |
| Political Orientation | -0.01 | [-0.24, 0.21] |  |
| Pol. Or. X Neutral vs. Left Right | 0.10 | [-0.37, 0.57] |  |
| Pol. Or. X Left vs. Right | -0.61* | [-1.16, -0.06] |  |
|  |  |  | *R^2^*  = .296** |
|  |  |  | 95% CI[.18,.38] |
|  |  |  |  |

*Note.* Linerar Regression using helmert coding. * indicates p < .05. ** indicates p < .01. The Moderator Political Orientation was z-transformed before the analysis.

**Results Analysis Picture 9**

We used a helmert coding to calculate the linear regression testing our hypothesis. The regression analysis showed a significant interaction between political orientation and the left-right conditions. A plot of the interaction (*Figure 1*) revealed the expected pattern.

**Figure 1:** Plot of the Interaction for Picture 9

**
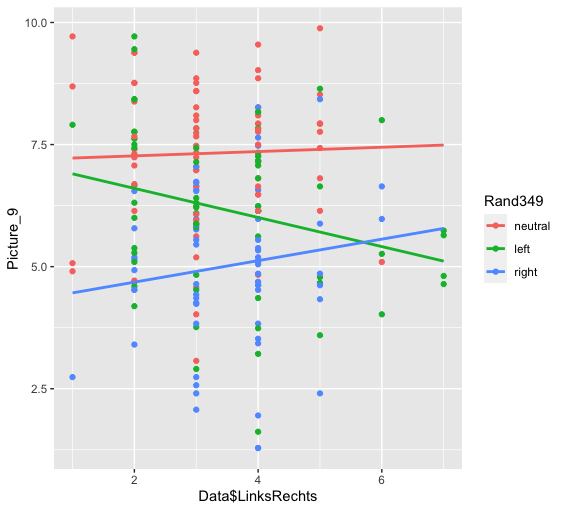
**
