# Supplementary material for: If graffiti changed anything, it would be illegal. The influence of political graffiti on the perception of neighborhoods and intergroup attitudes
Source: Front Psychol. 2023 Jul 13;14:1098105. doi: 10.3389/fpsyg.2023.1098105 (PMC10403061; doi:10.3389/fpsyg.2023.1098105)
Supplement: Supplementary file 2 [file Data_Sheet_2.docx]

**Supplemental Materials (S2) for: If Graffiti Changed Anything, It Would Be Illegal. The Influence of Political Graffiti on the Perception of Neighborhoods and Intergroup Attitudes.**

**Study 2:**

[Exploratory Factor Analysis Neighborhood 1 2](#_Toc109648692)

[Pictures of the first Neighborhood 2](#_Toc109648693)

[Exploratory Factor Analysis 2](#_Toc109648694)

[Confirmatory Factor Analysis Neighborhood 2 & 3 6](#_Toc109648695)

[Confirmatory Factor Analysis 7](#_Toc109648696)

[Repeated Measure ANOVA 9](#_Toc109648697)

[Evaluation of Inhabitants 10](#_Toc109648698)

[Cohesion 11](#_Toc109648699)

[Social Control 12](#_Toc109648700)

[Exploratory Analysis: Acculturation Attitudes 13](#_Toc109648701)

# Exploratory Factor Analysis Neighborhood 1

## Pictures of the first Neighborhood

**Figure S2.1**: Overview of Picutres in Neighborhood 1

**
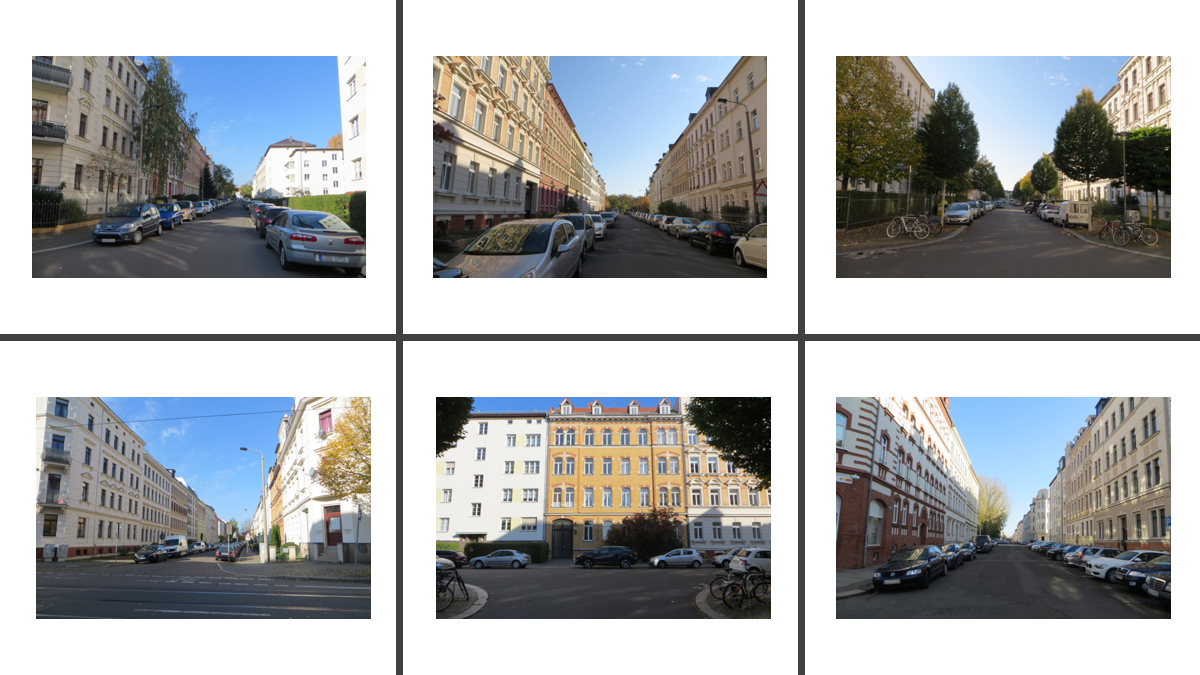
**

Method: Each picture was displayed for 5 Seconds individually in full screen. Depicted in Figure S2.1 is the overview of the pictures that were presented while participants were asked to fill out the questions (method adapted from O’Brien & Wilson, 2011).

## Exploratory Factor Analysis

As described in our preregistration, we used an EFA to identify the factor structure and suitable items from our item pool in neighborhood 1. For the EFA we used the open-source software JAMOVI (The Jamovi Project, 2021). The Dataset can be downloaded from the OSF repository.

In a first step, we entered all 18 items into the EFA. Bartlett’s test of sphericity was significant, χ2 (153) = 904, *p* < .001, indicated that correlations between items were sufficiently large for EFA. All KMO values for individual items were > .65, which is well above the acceptable limit of .5 (Field, 2009). We used principal axis factoring and a promax rotation and for the number of factor extraction we used parallel analysis. Factor loadings are reported in Table S2.1 for all items.

**Table S2.1**: Factor loadings for all Items in the Exploratory Factor Analysis

|  |  | **Factor** | | | |
| --- | --- | --- | --- | --- | --- |
| **Item** | **Wording** | **1** | **2** | **3** | **4** |
| COH_1 | This is a close-knit neighborhood. | -0.20 | -0.02 | **0.63** | 0.06 |
| COH_2 | People in this neighborhood generally don’t get along with each other. (r) | -0.01 | 0.21 | -0.02 | **0.42** |
| COH_3 | People in this neighborhood share the same values. | 0.04 | **0.41** | 0.27 | -0.02 |
| COH_4 | People around here are willing to help their neighbors. | 0.04 | 0.18 | **0.52** | 0.04 |
| COH_5 | People in this neighborhood mistrust each other. (r) | -0.18 | 0.16 | 0.11 | **0.59** |
| CON_1 | Inhabitants of this area would do something for the neighborhood. | 0.37 | -0.24 | 0.32 | 0.13 |
| CON_2 | Inhabitants would act against social injustices in the neighborhood. | **0.63** | -0.02 | 0.03 | -0.10 |
| CON_3 | Inhabitants would be active in a neighborhood organization. | 0.21 | -0.05 | 0.22 | 0.23 |
| CON_4 | Inhabitants would act if a fight broke out in front of their house. | **0.57** | 0.10 | -0.04 | 0.03 |
| CON_5 | Inhabitants would talked about events or problems with neighbors. | **0.58** | 0.04 | -0.12 | 0.17 |
| CON_6 | Inhabitants would do something in your free time together with your neighbors (e.g. drink coffee, have a barbecue, do sports) | 0.21 | -0.16 | 0.11 | **0.43** |
| CON_7 | Inhabitants would borrow something from your neighbors (e.g. tools, food). | **0.73** | -0.08 | -0.15 | -0.01 |
| CON_8 | Inhabitants would approach troublemakers. | **0.69** | 0.15 | -0.06 | -0.24 |
| SMC_1 | People in this neighborhood can be trusted. | -0.04 | **0.66** | 0.13 | 0.00 |
| SMC_2 | Most people in this area are unfriendly. (r) | 0.07 | **0.54** | 0.23 | -0.10 |
| SMC_3 | If you were in trouble, there are lots of people in this area who would help you. | 0.11 | **0.72** | 0.03 | 0.03 |
| SMC_4 | Tehre are adults in this neighborhood that children can look up to. | -0.04 | **0.62** | -0.22 | 0.17 |
| SMC_5 | This is a dangerous neighborhood. (r) | 0.00 | **0.49** | -0.21 | 0.19 |
| ***Note:*** 'Principal axis factoring' extraction method was used in combination with a 'promax' rotation. Factor loadings above .4 are in bold. | | | | | |

The EFA using parallel analysis revealed a 4-factor structure. (Eigenvalue Progression: 2.71, 2.14, 0.63, 0.36…). Except three items (CON_1, CON_3, CON_6) our items for *Social Control* loaded on the first factor with loadings above .5 – representing the ability to enforce social control in the neighborhood; Item 7 falls out of this interpretation. The items concerning the *stereotypes about* *inhabitants of the area* show loadings above .5 or close to .5 on the second factor. The items for *Social Cohesion* show loadings on several factors. Item loading on factors three and four however represent positive and negative wording in the items pool. Both factors correlated strongly with *r* = .51.

In the second step, we dropped items with low loadings (< .05) or cross loadings and selected those most suitable to represent our measured construct. Loadings and factor structure of the EFA containing those selected items can be seen in Table 2. The Scree plot indicated a 3-factor solution (Eigenvalue progression: 2.07, 1.30, 0.50, 0.22 …). Factor one represents evaluations about the inhabitants and explained 14.6 % of the variance. Factor two represents the ability to enforce social control and explained an additional 11.2% of the variance. The third factor explained 8.3 % of the variance and represented the preceded social cohesion in the neighborhood. 34.2 % of the total variance were explained.

**Table 2.2:** Factor Structure of the Scale after Removing Items with Low and Cross Loadings

|  |  |  |  |  |
| --- | --- | --- | --- | --- |
|  |  | **Factor** | | |
| **Item** | **Item Wording** | **1** | **2** | **3** |
| *1. factor: Evaluation of inhabitants (ST)* | |  |  |  |
| SMC_5r | This is a dangerous neighborhood. (rev) | **0.76** | -0.05 | -0.14 |
| SMC_4 | There are adults in this neighborhood that children can look up to. | **0.71** | 0.14 | -0.04 |
| SMC_1 | People in this neighborhood can be trusted. | **0.61** | 0.00 | 0.07 |
| SMC_2r | Most people in this area are unfriendly. (rev) | **0.55** | -0.03 | -0.04 |
| *2. factor: social control (SCon)* | |  |  |  |
| CON_8 | Inhabitants would approach troublemakers. | 0.04 | **0.63** | -0.15 |
| CON_2 | Inhabitants would act against social injustices in the neighborhood. | -0.03 | **0.59** | -0.06 |
| CON_4 | Inhabitants would act if a fight broke out in front of their house. | 0.03 | **0.57** | 0.08 |
| CON_5 | Inhabitants would talk about events or problems with neighbors. | 0.01 | **0.56** | 0.13 |
| *3. factor: social cohesion (SCoh)* | |  |  |  |
| COH_5r | People in this neighborhood mistrust each other. (rev) | 0.22 | -0.16 | **0.53** |
| COH_1 | This is a close-knit neighborhood. | -0.14 | -0.09 | **0.52** |
| CON_6 | ... do something in your free time together with your neighbors (e.g. drink coffee, have a barbecue, do sports) | -0.14 | 0.17 | **0.44** |
| COH_4 | People around here are willing to help their neighbors. | 0.08 | 0.10 | **0.39** |
| COH_2r | People in this neighborhood generally don’t get along with each other. (rev) | 0.25 | 0.00 | **0.34** |
| *Note.* 'Principal axis factoring' extraction method was used in combination with a 'promax' rotation. | | | |  |

# Confirmatory Factor Analysis Neighborhood 2 & 3

**Figure S2.2:** Overview of Pictures in Neighborhood 2


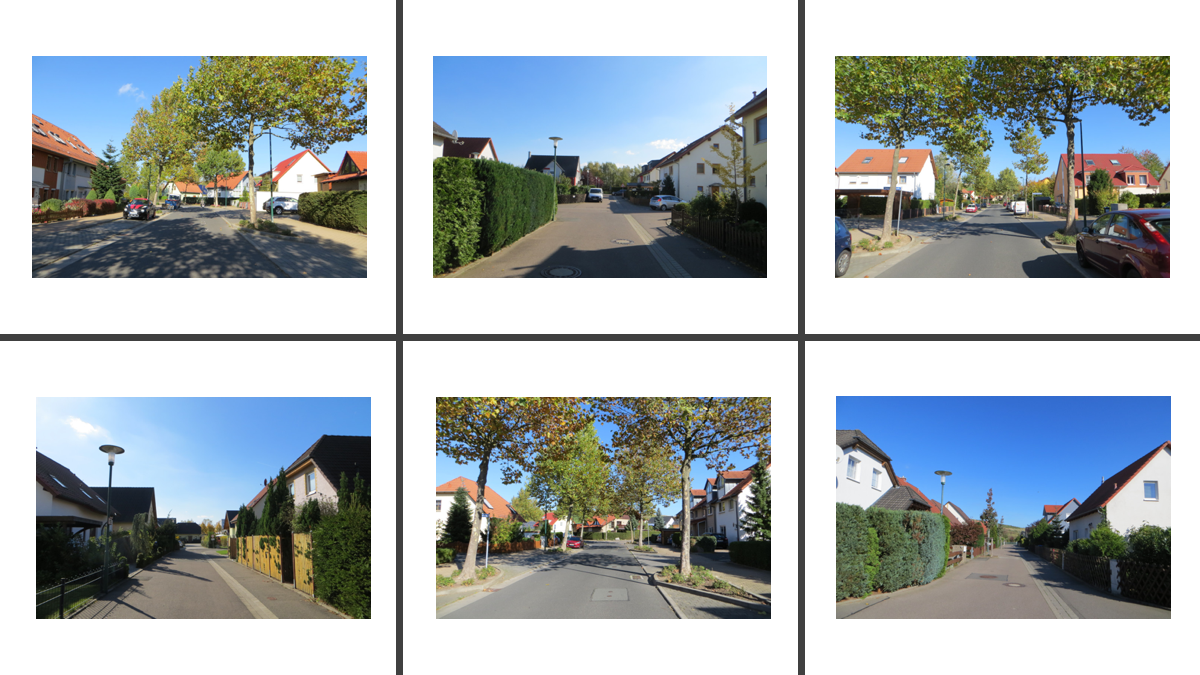


**Figure S2.3:** Overview of Pictures in Neighborhood 3


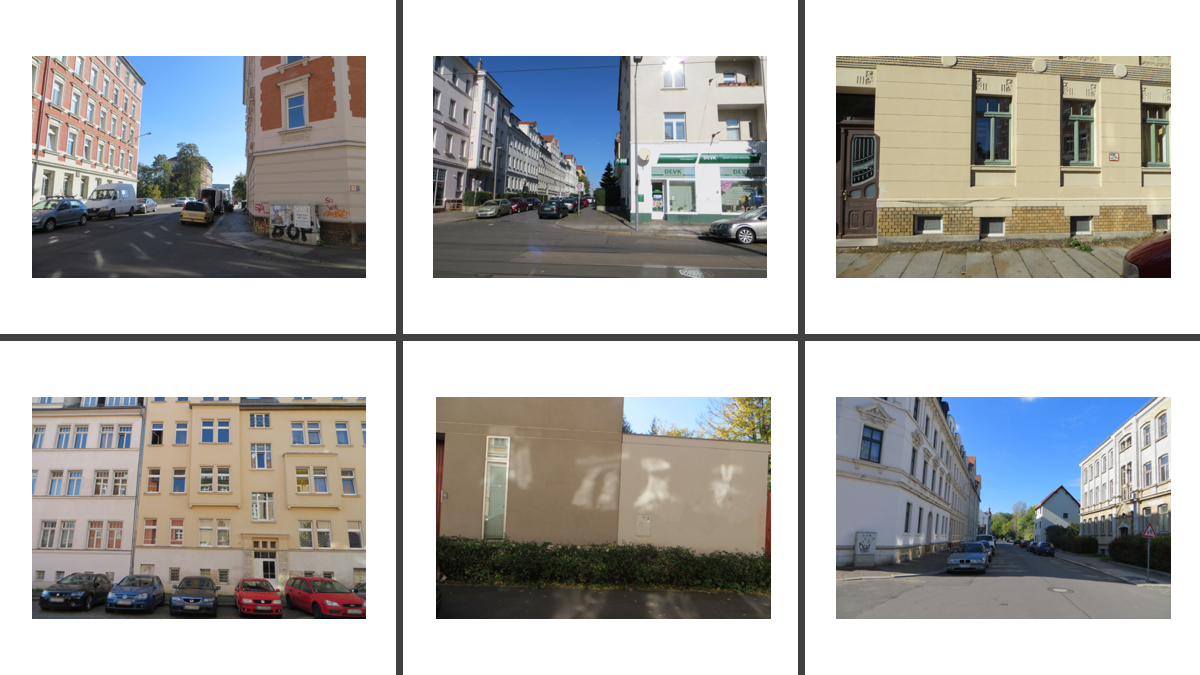


## Confirmatory Factor Analysis

In the next step, we used a Confirmatory Factor (CFA) analysis to verify the found structure in the EFA with each of the three presented neighborhoods. We removed one item from the social cohesion scale (CON_6) since it was assessed on a reversed scale and showed strong residual covariances with the other items of the assessed block. Further, we added residual covariances to those items in a scale, that were reversed to account for positive and negative worded items as a method factor. Also, factor loadings for items from the social cohesion showed low loadings < .5. Factor loadings are reported in Table S2.3 and S2.4 Overall, the CFA supported the found factor structure from the EFA. Fit indices and descriptive statistics are reported in Table S2.5 and S2.6.

**Table S2.3:** Confirmatory Factor Analysis Neighborhood 2

| **Factor** | **Item** | **Item Wording** | **Estimate** | **SE** | **Z** | **p** |
| --- | --- | --- | --- | --- | --- | --- |
| 1.Evaluation of Inhabitants | SMCWG3_5r | This is a dangerous neighborhood. (rev) | 0.801 | 0.085 | 9.455 | < .001 |
|  | SMCWG3_2r | There are adults in this neighborhood that children can look up to. | 0.772 | 0.081 | 9.546 | < .001 |
|  | SMCWG3_4 | People in this neighborhood can be trusted. | 1.086 | 0.083 | 13.074 | < .001 |
|  | SMCWG3_1 | Most people in this area are unfriendly. (rev) | 0.957 | 0.083 | 11.488 | < .001 |
| 2. Social Control | CONWG3_8 | Inhabitants would approach troublemakers. | 0.503 | 0.145 | 3.475 | < .001 |
|  | CONWG3_5 | Inhabitants would act against social injustices in the neighborhood. | 0.329 | 0.134 | 2.462 | 0.014 |
|  | CONWG3_4 | Inhabitants would act if a fight broke out in front of their house. | 0.457 | 0.178 | 2.559 | 0.01 |
|  | CONWG3_2 | Inhabitants would talk about events or problems with neighbors. | 0.524 | 0.15 | 3.481 | < .001 |
| 3. Social Cohesion | COHWG3_4 | People in this neighborhood mistrust each other. (rev) | 1.511 | 0.108 | 14.027 | < .001 |
|  | COHWG3_1 | This is a close-knit neighborhood. | 1.238 | 0.101 | 12.204 | < .001 |
|  | COHWG3_2r | People around here are willing to help their neighbors. | 1.653 | 0.102 | 16.238 | < .001 |
|  | COHWG3_5r | People in this neighborhood generally don’t get along with each other. (rev) | 1.473 | 0.1 | 14.673 | < .001 |

**Table S2.4:** Confirmatory Factor Analysis Neighborhood 3

| **Factor** | **Item** | **Item Wording** | **Estimate** | **SE** | **Z** | **p** |
| --- | --- | --- | --- | --- | --- | --- |
| 1.Evaluation of Inhabitants | SMCWG3_5r | This is a dangerous neighborhood. (rev) | 0.991 | 0.087 | 11.434 | < .001 |
|  | SMCWG3_2r | There are adults in this neighborhood that children can look up to. | 0.645 | 0.08 | 8.056 | < .001 |
|  | SMCWG3_4 | People in this neighborhood can be trusted. | 0.819 | 0.073 | 11.249 | < .001 |
|  | SMCWG3_1 | Most people in this area are unfriendly. (rev) | 0.639 | 0.062 | 10.357 | < .001 |
| 2. Social Control | CONWG3_8 | Inhabitants would approach troublemakers. | 1.012 | 0.098 | 10.367 | < .001 |
|  | CONWG3_5 | Inhabitants would act against social injustices in the neighborhood. | 0.776 | 0.087 | 8.881 | < .001 |
|  | CONWG3_4 | Inhabitants would act if a fight broke out in front of their house. | 1.122 | 0.087 | 12.951 | < .001 |
|  | CONWG3_2 | Inhabitants would talk about events or problems with neighbors. | 0.929 | 0.089 | 10.419 | < .001 |
| 3. Social Cohesion | COHWG3_4 | People in this neighborhood mistrust each other. (rev) | 0.547 | 0.078 | 6.981 | < .001 |
|  | COHWG3_1 | This is a close-knit neighborhood. | 0.434 | 0.084 | 5.155 | < .001 |
|  | COHWG3_2r | People around here are willing to help their neighbors. | 0.427 | 0.081 | 5.297 | < .001 |
|  | COHWG3_5r | People in this neighborhood generally don’t get along with each other. (rev) | 0.629 | 0.084 | 7.489 | < .001 |

**Table S2.5:** Fit indices for confirmatory factor analysis for each Neighborhood

|  |  |  |  |  |  |
| --- | --- | --- | --- | --- | --- |
|  |  |  |  | RMSEA 90% CI | |
|  | CFI | TLI | RMSEA | Lower | Upper |
| Neighborhood 1 | .96 | .95 | .04 | .00 | .06 |
| Neighborhood 2 | .97 | .96 | .06 | .03 | .08 |
| Neighborhood 3 | .97 | .96 | .05 | .02 | .07 |

**Table S2.6.** Scale Reliability, Means, Standard Deviations and Intercorrelations between the Scales

|  |  |  |  | **Correlations** | |
| --- | --- | --- | --- | --- | --- |
|  |  | *M (SD)* | α | 1. | 2. |
| Area 1 | 1. Stereotypes | 5.23 (0.89) | .74 |  |  |
|  | 2. Social Control | 4.22 (1.05) | .67 | -.01 |  |
|  | 3. Social Cohesion | 4.64 (0.72) | .56 | .31^**^ | .13^*^ |
| Area 2 | 1. Stereotypes | 5.33 (0.99) | .80 |  |  |
|  | 2. Social Control | 5.04 (1.55) | .89 | .53^**^ |  |
|  | 3. Social Cohesion | 5.49 (0.88) | .83 | -.05 | .10 |
| Area 3 | 1. Stereotypes | 4.46 (0.87) | .75 |  |  |
|  | 2. Social Control | 3.75 (1.08) | .79 | .34^**^ |  |
|  | 3. Social Cohesion | 3.85 (0.75) | .61 | .66^**^ | .32^**^ |

Note: * p < .05; ** p < 0.01

# Repeated Measure ANOVA

We calculated repeated measures ANOVAs to investigate if the scales were capable to detect differences between the neighborhoods. We report Tukeys HSD Post Hoc comparison.

## Evaluation of Inhabitants

**Table S2.7:** Repeated Measures ANOVA for Evaluation of Inhabitants

|  | **Sphericity Correction** | **Sum of Squares** | **df** | **Mean Square** | **F** | **p** | **η²** |
| --- | --- | --- | --- | --- | --- | --- | --- |
| RM Factor | None | 97.783 | 2 | 48.892 | 72.002 | < .001 | 0.154 |
|  | Huynh-Feldt | 97.783 | 1.984 | 49.297 | 72.002 | < .001 | 0.154 |
| Residual | None | 286.55 | 422 | 0.679 |  |  |  |
|  | Huynh-Feldt | 286.55 | 418.5 | 0.685 |  |  |  |

Note: Type 3 Sums of Squares

**Table S2.8:** Tukey Post-Hoc Comparison for Evaluation of Inhabitants

| **Comparison** | | |  |  |  |  |  |
| --- | --- | --- | --- | --- | --- | --- | --- |
| **RM Factor** |  | **RM Factor** | **Mean Difference** | **SE** | **df** | **t** | **p** |
| Neighborhood 1 | - | Neighborhood 2 | -0.1 | 0.08 | 422 | -1.252 | 0.423 |
|  | - | Neighborhood 3 | 0.777 | 0.08 | 422 | 9.71 | < .001 |
| Neighborhood 2 | - | Neighborhood 3 | 0.877 | 0.08 | 422 | 10.96 | < .001 |

Note: Tukey corrected p-values

**Figure S2.1**: Results of the RM ANOVA for Evaluation of Inhabitants


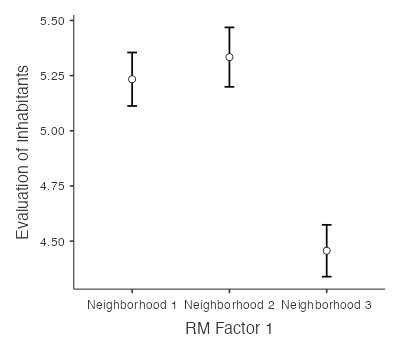


Note: Means and 95% Confidence Intervals are depicted. Scale range is 1-7.

## Cohesion

**Table S2.7:** Repeated Measures ANOVA for Cohesion

|  |  |  |  |  |  |  |  |
| --- | --- | --- | --- | --- | --- | --- | --- |
|  | **Sphericity Correction** | **Sum of Squares** | **df** | **Mean Square** | **F** | **p** | **η²** |
| RM Factor | None | 287.37 | 2 | 143.685 | 265.89 | < .001 | 0.424 |
|  | Huynh-Feldt | 287.37 | 1.89 | 152.087 | 265.89 | < .001 | 0.424 |
| Residual | None | 228.046 | 422 | 0.54 |  |  |  |
|  | Huynh-Feldt | 228.046 | 398.7 | 0.572 |  |  |  |
|  |  |  |  |  |  |  |  |

Note: Type 3 Sums of Squares

**Table S2.8:** Tukey Post-Hoc Comparison for Cohesion

| **Comparison** | | |  |  |  |  |  |
| --- | --- | --- | --- | --- | --- | --- | --- |
| **RM Factor** |  | **RM Factor** | **Mean Difference** | **SE** | **df** | **t** | **p** |
| Neighborhood 1 | - | Neighborhood 2 | -0.85 | 0.071 | 422 | -11.91 | < .001 |
|  | - | Neighborhood 3 | 0.796 | 0.071 | 422 | 11.15 | < .001 |
| Neighborhood 2 | - | Neighborhood 3 | 1.646 | 0.071 | 422 | 23.06 | < .001 |

Note: Tukey corrected p-values

**Figure S2.2:** Results of the RM ANOVA for Cohesion


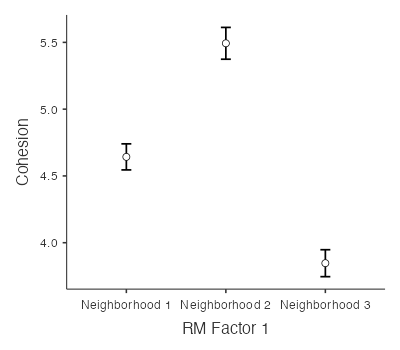


Note: Means and 95% Confidence Intervals are depicted. Scale range is 1-7.

## Social Control

**Table S2.9:** Repeated Measures ANOVA for Control

|  | **Sphericity Correction** | **Sum of Squares** | **df** | **Mean Square** | **F** | **p** | **η²** |
| --- | --- | --- | --- | --- | --- | --- | --- |
| RM Factor | None | 181.374 | 2 | 90.687 | 68.635 | < .001 | 0.155 |
|  | Huynh-Feldt | 181.374 | 1.632 | 111.168 | 68.635 | < .001 | 0.155 |
| Residual | None | 557.584 | 422 | 1.321 |  |  |  |
|  | Huynh-Feldt | 557.584 | 344.3 | 1.62 |  |  |  |

Note: Type 3 Sums of Squares

**Table 2.10:** Tukey Post-Hoc Comparison for Control

| **Comparison** | | |  |  |  |  |  |
| --- | --- | --- | --- | --- | --- | --- | --- |
| **RM Factor** |  | **RM Factor** | **Mean Difference** | **SE** | **df** | **t** | **p** |
| Neighborhood 1 | - | Neighborhood 2 | -0.827 | 0.112 | 422 | -7.404 | < .001 |
|  | - | Neighborhood 3 | 0.465 | 0.112 | 422 | 4.162 | < .001 |
| Neighborhood 2 | - | Neighborhood 3 | 1.291 | 0.112 | 422 | 11.57 | < .001 |

Note: Tukey corrected p-values

**Figure S2.3:** Results of the RM ANOVA for Control


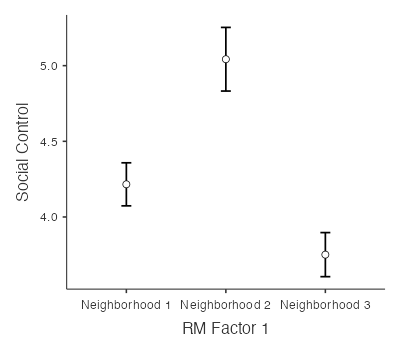


Note: Means and 95% Confidence Intervals are depicted. Scale range is 1-7.

## Exploratory Analysis: Acculturation Attitudes

We added one measure to test how participants would rate the acculturation attitudes of the inhabitants of the neighborhoods. We employed the same analysis strategy as in the previous RM ANOVAs.

**Table S2.11:** Repeated Measures ANOVA for Acculturation Attitudes

|  | **Sphericity Correction** | **Sum of Squares** | **df** | **Mean Square** | **F** | **p** | **η²** |
| --- | --- | --- | --- | --- | --- | --- | --- |
| RM Factor | None | 62.55 | 2 | 31.275 | 31.667 | < .001 | 0.073 |
|  | Huynh-Feldt | 62.55 | 1.953 | 32.032 | 31.667 | < .001 | 0.073 |
| Residual | None | 416.783 | 422 | 0.988 |  |  |  |
|  | Huynh-Feldt | 416.783 | 412 | 1.012 |  |  |  |

Note: Type 3 Sums of Squares

**Table 2.11:** Tukey Post-Hoc Comparison for Acculturation Attitudes

| **Comparison** | | |  |  |  |  |  |
| --- | --- | --- | --- | --- | --- | --- | --- |
| **RM Factor** |  | **RM Factor** | **Mean Difference** | **SE** | **df** | **t** | **p** |
| Neighborhood 1 | - | Neighborhood 2 | 0.75 | 0.093 | 211 | 8.025 | < .001 |
|  | - | Neighborhood 3 | 0.231 | 0.091 | 211 | 2.549 | 0.031 |
| Neighborhood 2 | - | Neighborhood 3 | -0.519 | 0.105 | 211 | -4.948 | < .001 |

Note: Tukey corrected p-values

**Figure S2.3:** Results of the RM ANOVA for Control


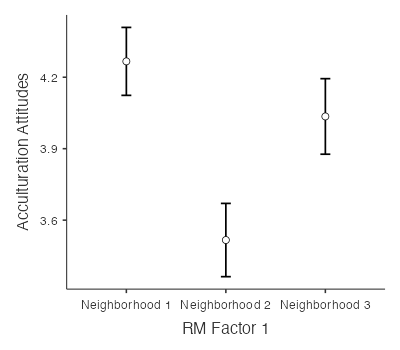


Note: Means and 95% Confidence Intervals are depicted. Scale range is 1-7.
