# Supplementary material for: If graffiti changed anything, it would be illegal. The influence of political graffiti on the perception of neighborhoods and intergroup attitudes
Source: Front Psychol. 2023 Jul 13;14:1098105. doi: 10.3389/fpsyg.2023.1098105 (PMC10403061; doi:10.3389/fpsyg.2023.1098105)
Supplement: Supplementary file 3 [file Data_Sheet_3.docx]

**Supplemental Materials (S3) for: If Graffiti Changed Anything, It Would Be Illegal. The Influence of Political Graffiti on the Perception of Neighborhoods and Intergroup Attitudes.**

**Study 3**

**Overview:**

We report descriptive statistics of the groups and Post-hoc Tukey HSD.

[Social Control 2](#_Toc109653521)

[Cohesion 3](#_Toc109653522)

[Evaluation of Inhabitants 4](#_Toc109653523)

[Exploartory: Feeling Thermometer 5](#_Toc109653524)

# Social Control

**Table 3.1:** Group Size, Mean and Standard Deviation of the Groups for Social Control

| **Group** | ***n*** | **Mean** | ***SD*** |
| --- | --- | --- | --- |
| Control Group | 120 | 3.83 | 0.08 |
| Group 1 | 105 | 3.46 | 0.10 |
| Group 2 | 106 | 3.30 | 0.12 |
| Group 3 | 98 | 3.14 | 0.14 |
| Group 4 | 125 | 3.33 | 0.12 |

**Table S3.2:** Post-hoc Tukey HSD with Social Control as Dependent Variable

|  |  |  |  |  |  |  |  |  | 95% CI | |
| --- | --- | --- | --- | --- | --- | --- | --- | --- | --- | --- |
|  |  |  | Mean Difference | SE | *df* | *t* | *p* | *d* | LL | UL |
| **1** | **-** | **2** | 0.37 | 0.16 | 549.00 | 2.32 | 0.139 | 0.31 | 0.05 | 0.57 |
|  | **-** | **3** | 0.53 | 0.16 | 549.00 | 3.32 | 0.008 | 0.44 | 0.18 | 0.71 |
|  | **-** | **4** | 0.69 | 0.16 | 549.00 | 4.23 | 0.000 | 0.58 | 0.31 | 0.85 |
|  | **-** | **5** | 0.50 | 0.15 | 549.00 | 3.29 | 0.009 | 0.42 | 0.17 | 0.67 |
| **2** | **-** | **3** | 0.16 | 0.16 | 549.00 | 0.96 | 0.872 | 0.13 | -0.14 | 0.40 |
|  | **-** | **4** | 0.32 | 0.17 | 549.00 | 1.89 | 0.322 | 0.27 | -0.01 | 0.54 |
|  | **-** | **5** | 0.13 | 0.16 | 549.00 | 0.83 | 0.920 | 0.11 | -0.15 | 0.37 |
| **3** | **-** | **4** | 0.16 | 0.17 | 549.00 | 0.95 | 0.876 | 0.13 | -0.14 | 0.41 |
|  | **-** | **5** | -0.03 | 0.16 | 549.00 | -0.17 | 1.000 | -0.02 | -0.28 | 0.24 |
| **4** | **-** | **5** | -0.19 | 0.16 | 549.00 | -1.15 | 0.779 | -0.16 | -0.42 | 0.11 |

Note: Tukey corrected p-values

# Cohesion

**Table 3.4:** Group Size, Mean and Standard Deviation of the Groups for Cohesion

| **Group** | ***n*** | **Mean** | ***SD*** |
| --- | --- | --- | --- |
| Control Group | 120 | 4.05 | 0.82 |
| Group 1 | 105 | 3.47 | 0.92 |
| Group 2 | 106 | 3.22 | 0.98 |
| Group 3 | 98 | 2.98 | 1.10 |
| Group 4 | 125 | 3.06 | 1.17 |

**Table S3.5:** Post-hoc Tukey HSD with Cohesion as Dependent Variable

|  |  |  |  |  |  |  |  |  | 95% CI | |
| --- | --- | --- | --- | --- | --- | --- | --- | --- | --- | --- |
|  |  |  | Mean Difference | SE | *df* | *t* | *p* | *d* | LL | UL |
| **1** | **-** | **2** | 0.58 | 0.13 | 549 | 4.31 | 0.000 | 0.58 | 0.31 | 0.84 |
|  | **-** | **3** | 0.82 | 0.13 | 549 | 6.12 | 0.000 | 0.82 | 0.55 | 1.08 |
|  | **-** | **4** | 1.07 | 0.14 | 549 | 7.78 | 0.000 | 1.06 | 0.78 | 1.33 |
|  | **-** | **5** | 0.99 | 0.13 | 549 | 7.66 | 0.000 | 0.98 | 0.72 | 1.24 |
| **2** | **-** | **3** | 0.24 | 0.14 | 549 | 1.75 | 0.404 | 0.24 | -0.03 | 0.51 |
|  | **-** | **4** | 0.49 | 0.14 | 549 | 3.44 | 0.006 | 0.48 | 0.21 | 0.76 |
|  | **-** | **5** | 0.41 | 0.13 | 549 | 3.05 | 0.020 | 0.40 | 0.14 | 0.67 |
| **3** | **-** | **4** | 0.24 | 0.14 | 549 | 1.73 | 0.415 | 0.24 | -0.03 | 0.52 |
|  | **-** | **5** | 0.16 | 0.13 | 549 | 1.23 | 0.731 | 0.16 | -0.10 | 0.42 |
| **4** | **-** | **5** | -0.08 | 0.14 | 549 | -0.59 | 0.976 | -0.08 | -0.34 | 0.19 |

Note: Tukey corrected p-values

# Evaluation of Inhabitants

**Table 3.6:** Group Size, Mean and Standard Deviation of the Groups for Evaluation of Inhabitants

| **Group** | ***n*** | **Mean** | ***SD*** |
| --- | --- | --- | --- |
| Control Group | 120 | 4.57 | 0.98 |
| Group 1 | 105 | 3.58 | 0.97 |
| Group 2 | 106 | 3.14 | 1.06 |
| Group 3 | 98 | 2.22 | 1.08 |
| Group 4 | 125 | 2.38 | 1.26 |

**Table S3.7:** Post-hoc Tukey HSD with Evaluation of Inhabitants as Dependent Variable

|  |  |  |  |  |  |  |  |  | 95% CI | |
| --- | --- | --- | --- | --- | --- | --- | --- | --- | --- | --- |
|  |  |  | Mean Difference | SE | *df* | *t* | *p* | *d* | LL | UL |
| **1** | **-** | **2** | 0.99 | 0.14 | 549 | 6.86 | 0.000 | 0.92 | 0.65 | 1.18 |
|  | **-** | **3** | 1.43 | 0.14 | 549 | 9.93 | 0.000 | 1.32 | 1.05 | 1.60 |
|  | **-** | **4** | 2.35 | 0.15 | 549 | 15.99 | 0.000 | 2.18 | 1.88 | 2.47 |
|  | **-** | **5** | 2.19 | 0.14 | 549 | 15.92 | 0.000 | 2.03 | 1.76 | 2.31 |
| **2** | **-** | **3** | 0.44 | 0.15 | 549 | 2.96 | 0.026 | 0.41 | 0.14 | 0.68 |
|  | **-** | **4** | 1.36 | 0.15 | 549 | 8.98 | 0.000 | 1.26 | 0.97 | 1.55 |
|  | **-** | **5** | 1.21 | 0.14 | 549 | 8.45 | 0.000 | 1.12 | 0.85 | 1.39 |
| **3** | **-** | **4** | 0.92 | 0.15 | 549 | 6.09 | 0.000 | 0.85 | 0.57 | 1.13 |
|  | **-** | **5** | 0.77 | 0.14 | 549 | 5.38 | 0.000 | 0.71 | 0.45 | 0.97 |
| **4** | **-** | **5** | -0.15 | 0.15 | 549 | -1.06 | 0.829 | -0.14 | -0.41 | 0.12 |

Note: Tukey corrected p-values

# Exploartory: Feeling Thermometer

**Table 3.8:** Group Size, Mean and Standard Deviation of the Groups for Evaluation of Inhabitants

| **Group** | ***n*** | **Mean** | ***SD*** |
| --- | --- | --- | --- |
| Control Group | 120 | 67.27 | 20.51 |
| Group 1 | 105 | 69.00 | 20.27 |
| Group 2 | 106 | 62.80 | 20.78 |
| Group 3 | 98 | 70.57 | 17.93 |
| Group 4 | 125 | 71.13 | 17.73 |

**Table S3.9:** Post-hoc Tukey HSD with Evaluation of Inhabitants as Dependent Variable

|  |  |  |  |  |  |  |  |  | 95% CI | |
| --- | --- | --- | --- | --- | --- | --- | --- | --- | --- | --- |
|  |  |  | Mean Difference | SE | *df* | *t* | *p* | *d* | LL | UL |
| **1** | **-** | **2** | -1.73 | 2.60 | 549 | 0.506 | 0.96 | -0.09 | -0.35 | 0.17 |
|  | **-** | **3** | 4.47 | 2.60 | 549 | 0.085 | 0.42 | 0.23 | -0.03 | 0.49 |
|  | **-** | **4** | -3.30 | 2.65 | 549 | 0.215 | 0.73 | -0.17 | -0.44 | 0.10 |
|  | **-** | **5** | -3.86 | 2.49 | 549 | 0.122 | 0.53 | -0.20 | -0.45 | 0.05 |
| **2** | **-** | **3** | 6.21 | 2.68 | 549 | 0.021 | 0.14 | 0.32 | 0.05 | 0.59 |
|  | **-** | **4** | -1.56 | 2.74 | 549 | 0.568 | 0.98 | -0.08 | -0.36 | 0.20 |
|  | **-** | **5** | -2.13 | 2.58 | 549 | 0.410 | 0.92 | -0.11 | -0.37 | 0.15 |
| **3** | **-** | **4** | -7.77 | 2.73 | 549 | 0.005 | 0.04 | -0.40 | -0.68 | -0.12 |
|  | **-** | **5** | -8.33 | 2.57 | 549 | 0.001 | 0.01 | -0.43 | -0.69 | -0.17 |
| **4** | **-** | **5** | -0.57 | 2.63 | 549 | 0.830 | 1.00 | -0.03 | -0.29 | 0.24 |

Note: Tukey corrected p-values
